# Decelerating Spread of West Nile Virus by Percolation in a Heterogeneous Urban Landscape

**DOI:** 10.1371/journal.pcbi.1002104

**Published:** 2011-07-28

**Authors:** Krisztian Magori, Waheed I. Bajwa, Sarah Bowden, John M. Drake

**Affiliations:** 1 Odum School of Ecology, University of Georgia, Athens, Georgia, United States of America; 2 Office of Vector Surveillance and Control, New York City Department of Health and Mental Hygiene, New York, New York, United States of America; University of Michigan and Howard Hughes Med. Inst., United States of America

## Abstract

Vector-borne diseases are emerging and re-emerging in urban environments throughout the world, presenting an increasing challenge to human health and a major obstacle to development. Currently, more than half of the global population is concentrated in urban environments, which are highly heterogeneous in the extent, degree, and distribution of environmental modifications. Because the prevalence of vector-borne pathogens is so closely coupled to the ecologies of vector and host species, this heterogeneity has the potential to significantly alter the dynamical systems through which pathogens propagate, and also thereby affect the epidemiological patterns of disease at multiple spatial scales. One such pattern is the speed of spread. Whereas standard models hold that pathogens spread as waves with constant or increasing speed, we hypothesized that heterogeneity in urban environments would cause decelerating travelling waves in incipient epidemics. To test this hypothesis, we analysed data on the spread of West Nile virus (WNV) in New York City (NYC), the 1999 epicentre of the North American pandemic, during annual epizootics from 2000–2008. These data show evidence of deceleration in all years studied, consistent with our hypothesis. To further explain these patterns, we developed a spatial model for vector-borne disease transmission in a heterogeneous environment. An emergent property of this model is that deceleration occurs only in the vicinity of a critical point. Geostatistical analysis suggests that NYC may be on the edge of this criticality. Together, these analyses provide the first evidence for the endogenous generation of decelerating travelling waves in an emerging infectious disease. Since the reported deceleration results from the heterogeneity of the environment through which the pathogen percolates, our findings suggest that targeting control at key sites could efficiently prevent pathogen spread to remote susceptible areas or even halt epidemics.

## Author Summary

Current theory of the spatial spread of pathogens predicts travelling waves at constant or increasing speed in homogeneous environments. However, in urban environments, increasing and often unregulated development produces a highly heterogeneous landscape. Such heterogeneity affects pathogens spread by insect vectors particularly, which typically have short dispersal distances. We hypothesized that high levels of heterogeneity can slow the spread of such pathogens, resulting in decelerating epidemic waves. We analysed the annual spread of West Nile virus (WNV) in New York City (NYC), using a dataset containing >1,000,000 records since the origin of the North American pandemic in 1999. Our analysis provides the first evidence of endogenous decelerating travelling waves in an emerging infectious disease. We found that WNV spread with decreasing speed in each season and rejected four alternative hypotheses to explain this deceleration. A mathematical model shows that high levels of heterogeneity can lead to such decelerating travelling waves. Interestingly, the level of heterogeneity in land-cover types associated with WNV-positive dead birds in NYC is of the order of magnitude required to produce decelerating travelling waves in the model. Consequently, we propose that control strategies targeting key sites may be effective at slowing WNV spread in NYC.

## Introduction

Urbanization, due to both population growth in cities and immigration from rural communities, now concentrates almost half of the global human population (3.3 out of 6.8 billion people) into urban centres where crowding promotes the spread of infectious diseases [1]. Transmission of vector-borne diseases in urban environments, particularly dengue fever and dengue haemorrhagic fever [2], malaria [3], yellow fever [4], [5], and West Nile virus (WNV) fever [6], is an increasingly important global health concern [7], [8]. Not all of the causes of recent increases in urban vector-borne disease are clear. The modified environments of cities have a direct effect on populations of arthropod vectors through environmental drivers such as temperature and water retention [9]. More subtly, urbanization may have structural effects on disease transmission systems. Specifically, urban environments are highly heterogeneous in the extent, degree, and distribution of environmental modifications. While this heterogeneity directly translates into varying levels of risk for the inhabitants of different areas, it may also affect the dynamical transmission systems through which the pathogen propagates [10], just as heterogeneity in ecological systems gives rise to novel patterns of diversity and persistence [11].

We hypothesized that environmental heterogeneity in urban environments gives rise to decelerating waves of infection due to the inhibition of local propagation in locations unfavourable for disease transmission. We emphasize that the decelerating waves we hypothesize are not due to environmental or temporal gradients in transmission, as has been described previously for a fungal pathogen infecting plants [12], but solely to endogenous dynamics influenced by spatial heterogeneity. By analogy to the theory of percolation in disordered media [13], we conjecture for such systems the existence of a critical fraction of sites which must be “transmission-promoting” for an introduced pathogen to propagate. These predictions differ qualitatively from the asymptotically constant and accelerating waves predicted by the theory of spread in homogeneous environments [14]–[16] and observed in other systems [17]–[19].

To test this hypothesis, we studied the spread of WNV in the region of its epicentre in New York City over the period since its emergence in North America [20], [21]. WNV is a single-stranded positive sense RNA virus belonging to the genus Flavivirus, family Flaviviridae, and can cause fatal meningitis and encephalitis in humans [22], [23]. Persistence of the virus is maintained by an enzootic cycle primarily involving ornithophilic mosquitoes of the genus *Culex* and passerine birds [24]. Humans and other mammals (*e.g.*, horses) are dead-end hosts which get infected by the bite of infectious mosquitoes (also predominantly from the genus *Culex*). Transmission risk is the highest towards the end of each WNV season, typically in late summer and early fall. WNV is currently the most widespread arbovirus in the world and is now the most prevalent vector-borne disease of humans in North America.

Then, to better understand the effect of habitat heterogeneity on epidemic spread, we developed a percolation model for WNV transmission. Percolation theory, which has been used previously to study the spread of pathogens on contact networks [25]–[27], concerns the distribution of connected clusters in a random graph as a representation of liquid transport in a heterogeneous medium [13]. The porosity of the idealized medium is characterized by a global parameter *p*, the proportion of open sites. An important theoretical property of heterogeneous media is the existence in a lattice of infinite extension of a critical point, *p*_c_, which must be exceeded for an infinite cluster of adjacent sites to exist [13]. In practice, in finite lattices of even very small extent, *p*_c_ is the threshold that must be exceeded for connectivity, *i.e.*, the frequency of open sites required for the system to “percolate”. Such open and closed sites of percolating media are analogous to the environmental properties that impede and promote the transmission of pathogens in heterogeneous landscapes. It follows that there will exist a critical point in the fraction of transmission promoting habitats for the propagation of pathogens in heterogeneous environments [28], [29].

## Results and Discussion

### Decelerating Spread

**Figure 1:**
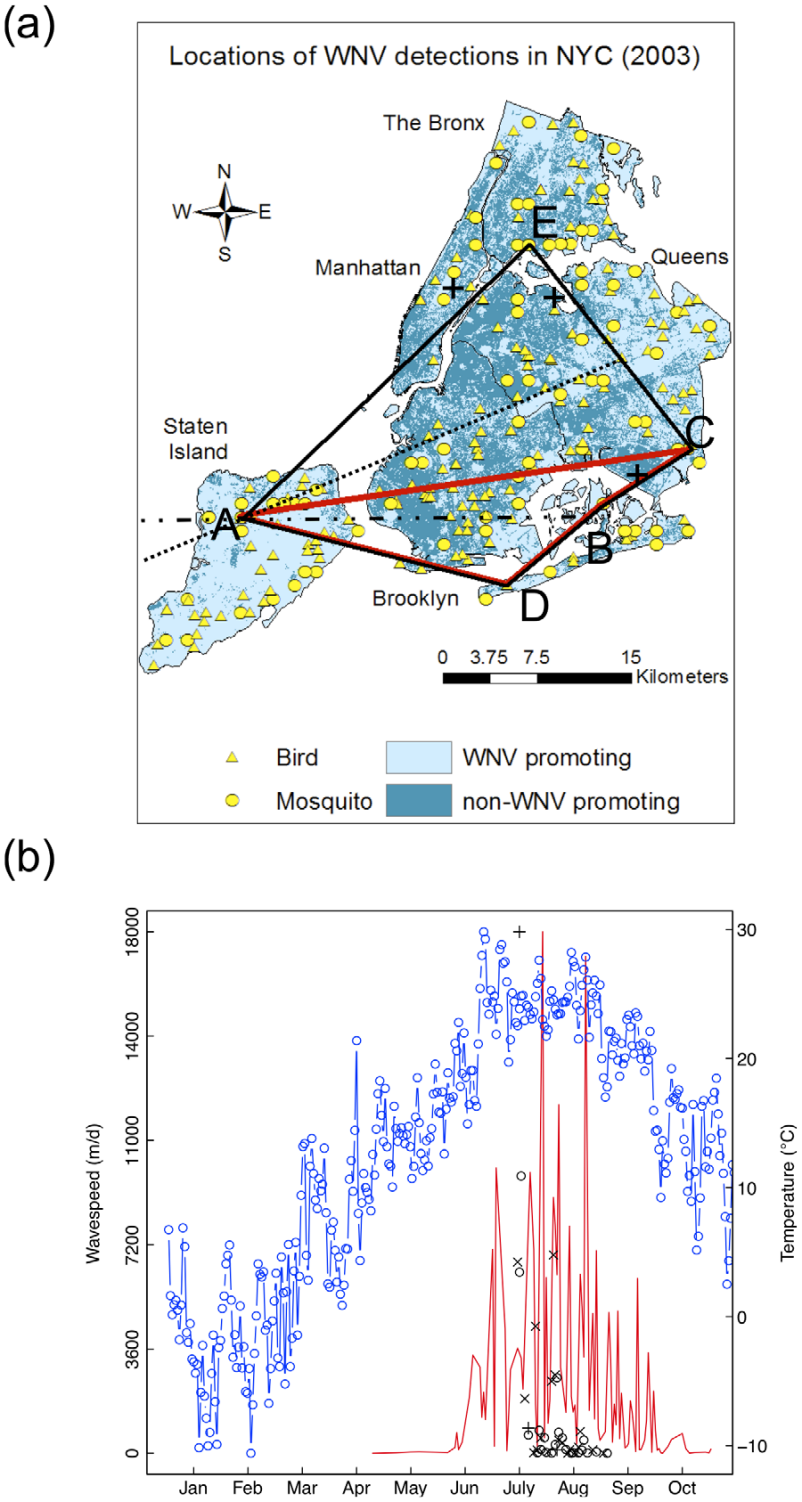
The spatial structure of annual WNV outbreaks in NYC, demonstrated for the year of 2003. (a) Speed of WNV spread was estimated from point locations of WNV-positive mosquito pools (circles) and WNV-positive dead birds (triangles). Dark and light cyan areas represent transmission-inhibiting and transmission-promoting land-cover types. The black crosses represent the approximate location of Central Park, La Guardia Airport and the John F. Kennedy International Airport respectively, where NOAA collects weather data. The first five locations where WNV was detected in 2003 are labelled as A,B,C,D and E, respectively. The first estimate of wave-speed was calculated using the convex hull method as (1) the increase of the square root area of the polygon encompassing ABCDE (black) relative to the square root area of the polygon encompassing ABCD (red) locations (convex hull method); (2) the difference between the average length of the two transects from A (ultrafine dashed and dash-dotted) intersecting the polygon ABCDE and the length of the transect (dash-dotted) intersecting the polygon ABCD (boundary displacement method); (3) the increase of the maximum distance between AC and AB (maximum distance method), divided by the time elapsed. Further evolution of the infected area can be seen on Fig. S1 in Text S1. (b) Estimated speed of WNV spread (black, in meters d^−1^), based on the convex hull method (circles), the boundary displacement method (symbol X ) and the maximum distance method (crosses), respectively; mean daily temperature (blue), and the total daily number of mosquitoes collected (red) over time.

Estimates of the speed at which waves of WNV spread across New York City during the years 2000–2008 ranged from 0.6 meters day^−1^ to 12 km day^−1^ using a method based on subsequent differences in the square root of the convex hull of observed infections (Fig. 1, Figs. S1,S2 and S3 in Text S1), 0.6 meters day^−1^ to 37 km day^−1^ using a maximum distance method, and 0.0000884 meters day^−1^ to 3.724 km day^−1^ using a boundary displacement method (Fig. 1)(see *Materials and Methods*). Changes in the estimated spread rate showed the hypothesized deceleration (negative correlation with time) in one or more analyses for all years (Table 1). In 2008, the virus appears to have originated from two separate locations giving rise to independent and converging wave fronts, compromising the detectability of deceleration using the convex hull and maximum distance methods. Thus, in contrast to the asymptotically constant wave-speeds predicted by theory for spread in a homogeneous environment [30], and the accelerating spread due to occasional long distance dispersal at the continental scale [31], the spread of WNV in New York City nearly always decelerated.

**Table 1:** Evidence for decelerating waves in WNV in NYC for 2000–2008.

	Year	Birds		Mosquito		Combined	
		ρ	p-value	ρ	p-value	ρ	p-value
*Convex hull*	2000	***−0.44***	***3.63×10^−8^***	**−0.236**	**0.0196**	***−0.461***	***9.16×10^−9^***
	2001	−0.11	0.141	**−0.225**	**0.0125**	***−0.260***	***3.98×10^−3^***
	2002	**−0.245**	**0.00353**	**−0.187**	**0.0353**	***−0.331***	***1.02×10^−4^***
	2003	***−0.374***	***5.93×10^−5^***	***−0.385***	***1.51×10^−4^***	***−0.497***	***4.66×10^−8^***
	2004	**−0.188**	**0.0397**	**−0.193**	**0.0238**	**−0.175**	**0.0336**
	2005	−0.0033	0.486	**−0.254**	**0.015**	−0.123	0.103
	2006	**−0.299**	**0.00596**	**−0.24**	**0.0101**	***−0.286***	***2.76×10^−3^***
	2007	−0.125	0.119	***−0.314***	***1.79×10^−3^***	***−0.371***	***1.94×10^−4^***
	2008			−0.145	0.0713		
*Maximum distance*	2000	**−0.234**	**0.00316**	−0.129	0.131	***−0.26***	***0.00114***
	2001	**−0.174**	**0.0437**	**−0.259**	**0.00484**	***−0.331***	***0.000314***
	2002	**−0.173**	**0.0296**	**−0.2085**	**0.0219**	**−0.231**	**0.00535**
	2003	**−0.26**	**0.0044**	**−0.254**	**0.00988**	**−0.24155**	**0.006985**
	2004	**−0.188**	**0.04**	−0.149	0.064	**−0.171**	**0.0368**
	2005	−0.0292	0.382	−0.0975	0.206	−0.04	0.3385
	2006	**−0.281**	**0.0198**	**−0.2125**	**0.02**	**−0.2125**	**0.0204**
	2007	**−0.197**	**0.031**	−0.147	0.09	−0.07575	0.238
	2008			−0.16	0.052		
*Boundary displacement*	2000	***−0.374***	***4.7×10^−6^***	***−0.317***	***0.00447***	***−0.401***	***8.39×10^−7^***
	2001	***−0.325***	***0.00197***	**−0.25**	**0.00699**	**−0.217**	**0.0163**
	2002	***−0.424***	***5.59×10^−6^***	***−0.297***	***0.00374***	***−0.441***	***9.73×10^−7^***
	2003	***−0.465***	***1.74×10^−6^***	***−0.336***	***0.001***	***−0.468***	***4.05×10^−7^***
	2004	−0.174	0.0662	***−0.3095***	***0.0015***	***−0.309***	***0.0012***
	2005	**−0.191**	**0.0442**	**−0.315**	**0.00503**	**−0.266**	**0.00607**
	2006	−0.156	0.111	**−0.218**	**0.0191**	**−0.269**	**0.005**
	2007	**−0.2435**	**0.0244**	***−0.416***	***8.325×10^−5^***	***−0.4415***	***1.31×10^−5^***
	2008			**−0.272**	**0.0059**		

P-values obtained from one-tailed hypothesis tests of Spearman's rank-order correlation between estimated wave speed and time elapsed. The extent of the infected area was estimated using three alternative methods: the convex hull encompassing all previous locations where WNV was detected, the average length of transects originating from the epicentre where they intersect these convex hulls, and the maximum Euclidean distance from the initial infected location (see Materials and Methods for details). The wave speed of WNV was calculated by dividing the increase of the extent of the infected area by the time elapsed since the last increase in the extent, for WNV-positive dead birds, WNV-positive mosquito pools, or the combination of both WNV-positive dead birds and mosquito pools. Values in bold italic are significant at the Holm-Bonferroni corrected level, while regular bold figures are significant at the nominal α = 0.05 level. The combined analysis, providing the greatest statistical power, provides evidence at the Holm-Bonferroni corrected level for a decelerating wave in 7 out of the 8 years studied, and for all years at the α = 0.05 level.

### Model

**Figure 2:**
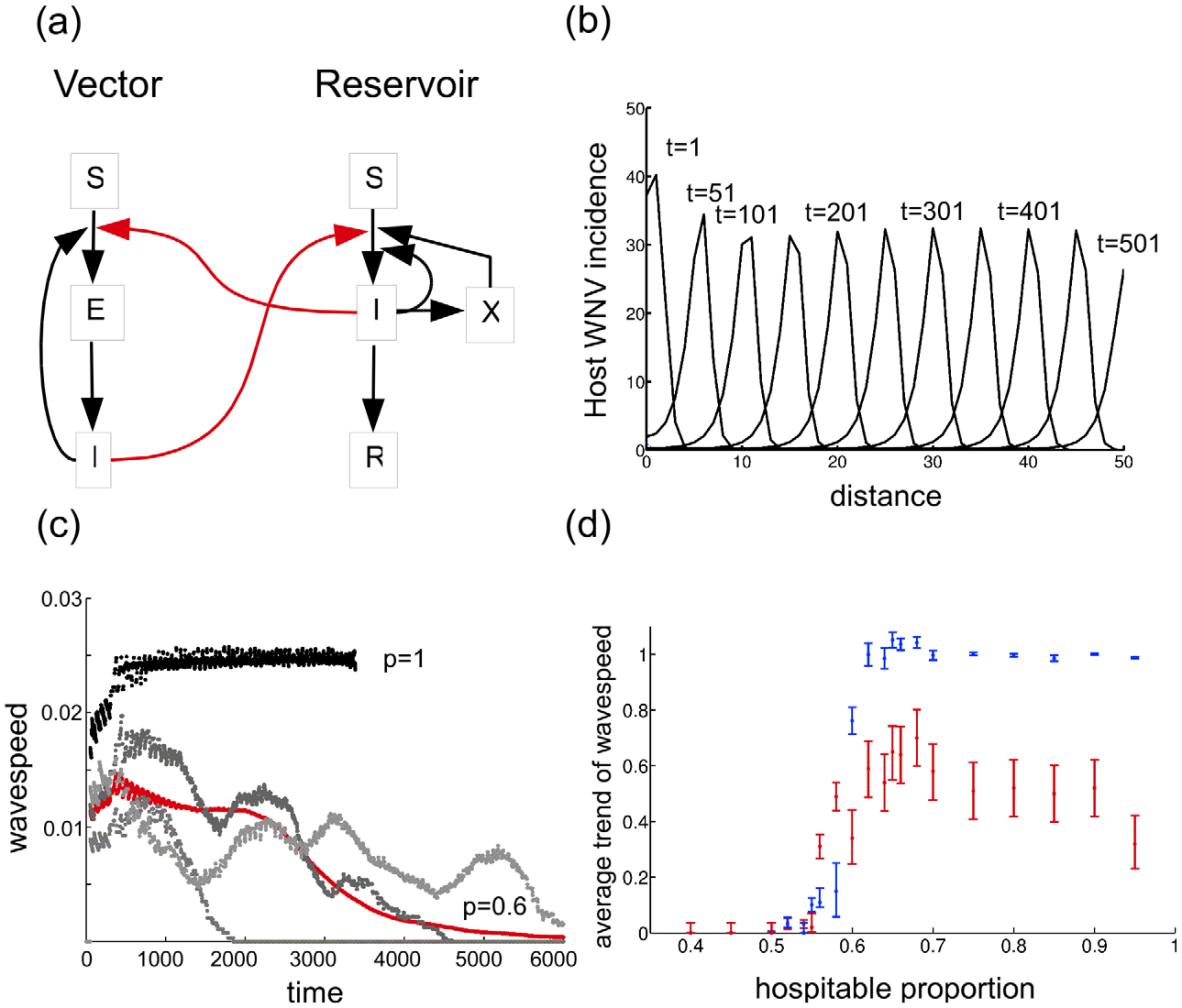
Transition from homogeneity to percolating phenomena in heterogeneous environments. (a) Compartmental model for local WNV transmission. Red and black arrows represent transmission routes incorporated into simplified and full models, respectively. The variable *X* represents dead birds that could be the source of infection to humans in a full model. (b) Travelling waves in the number of infectious hosts in homogeneous case (*p* = 1) from the origin along a tangent. (c) Time-dependent estimates of wave speed of the pathogen at *p* = 1 (black line) and at *p* = 0.6 (grey and red lines show individual runs and average, respectively). (d) Average trend of wave-speed (blue) and number of realizations where this trend is above 1 (red). Error bars show SE and 95% C.I. for the average trend and for the number of realizations, respectively.

**Figure 3:**
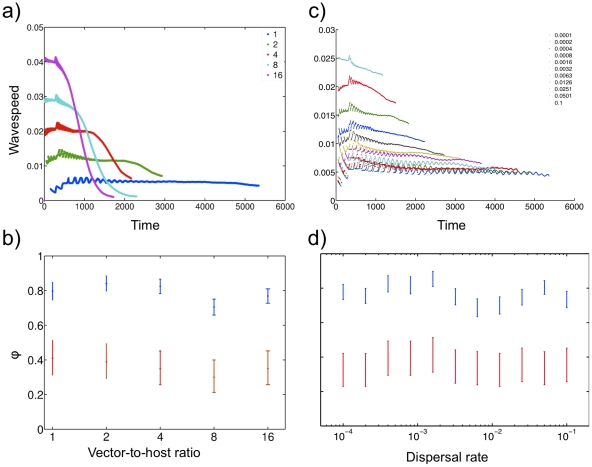
Sensitivity analysis of the wave speed to the ratio of vectors to hosts (*m* = *N*_V_/*N*_R_), and the local dispersal rate of hosts, in a heterogeneous lattice (*p* = 0.6). 100 simulations at each parameter value were performed on a lattice over 6,000 time steps. Parameters used were those listed in Table 2. (a) Average wave-speed was calculated as the first difference of the square root area of the convex hull (encompassing all previous sites where infectious hosts exceeded the threshold value of 1%) smoothed by a moving average with a wave-length of 500 time steps. *R*_0_ was calculated as 1.4, 2.0, 2.8, 3.9, and 5.6 for the vector-to-host ratios of 1, 2, 4, 8 and 16, respectively. Local dispersal rate was 0.01. (b) Average wave-speed trend (ratio of final and median wave-speed ; blue with error-bars representing the standard error) and frequency of realizations with (red with error bars representing 95% confidence intervals) at multiple vector-to-host ratios. The abscissa is on the log-scale. (c) Time-dependent estimates of wave speed of the pathogen at *p* = 0.6 at multiple dispersal rates. The constant ratio of vectors to hosts was set to 1. (d) Average trend of the final and the median wave-speed at multiple dispersal rates, with error bars showing standard error. Proportion of realizations with , with error bars showing the 95% confidence interval.

These patterns are well illustrated by our model (Fig. 2a). The basic reproductive number for the local dynamics given by our model was obtained using the “spectral radius method”, and is given by the expressionwhere and are the probability of transmission from an infectious vector to a reservoir host, and from an infectious reservoir host to a vector, respectively; is the biting rate of vectors; and are the length of the incubation period in the vectors and in the reservoir hosts, respectively; and are the mortality rates of the vectors and reservoir hosts; is the recovery rate of infectious reservoir hosts; is the excess mortality rate of infectious reservoir hosts; and are the total population size of vectors and reservoir hosts, respectively. Based on empirical measurements of these rates, our model predicts a local *R*_0_ for WNV between 1.4 and 4.4 for a vector-to-host ratio of 1 and 10, respectively (Fig. 3a). The expression for the basic reproductive number we present above is the square root of the “next generation” reproduction number, which assumes that the pathogen must pass through both the vector and the host. Although field-based estimates of the basic reproduction number are not available for West Nile Virus, our predictions are consistent with values estimated for other members of the *Flaviviridae* family [*e.g.*, 32]. Modelled patterns of spatial spread quantitatively elaborate on the qualitative pattern we predicted. Particularly, in a constant lattice with all sites promoting transmission, spread occurred according to a travelling wave with asymptotically constant speed following a transient increase and constant wave form, recovering the well known behaviour of spread in a homogeneous environment as a limiting case [14] (Fig. 2b). In heterogeneous habitats, however, as the fraction of transmission-promoting sites decreased, spatial spread of the pathogen was increasingly inhibited. One effect of heterogeneity was to diminish the eventual wave speed ultimately achieved relative to the homogeneous lattice (Fig. 2c). In the vicinity of the percolation threshold (*p*_c_ = 0.5927… for the von Neumann lattice used here [12]), another effect emerged: time series of observed spread rates were erratic, segmenting into periods of temporary acceleration and deceleration (troughs and peaks in Fig. 2c), due to alternating confinement of spread to narrow corridors and expansion in self-organized clusters of transmission-promoting sites. Finally, the aggregation of these accelerating and decelerating episodes resulted in a third effect (our main hypothesis): overall decelerating spread as the proportion of transmission-promoting sites decreased toward the critical point in its vicinity (0.52<*p*<0.6) (Fig. 2d).

To better understand the robustness of these patterns we further studied the sensitivity of wave speed to a variety of assumptions. First, we investigated the effect of variation in the vectors-to-host ratio (Fig. 3a). Necessarily, at or below the critical ratio of vectors to hosts, the pathogen did not spread in the lattice and immediately above this critical ratio, the wave-speed was too small to be measurable. However, further increasing the ratio of vectors to hosts lead to measurable wave speeds that increased with increasing vector-to-host ratio. Most importantly, the wave decelerated for a large range of vector-to-host ratios with no major differences between the average ratio of final and median wave-speed , a summary measure of deceleration, or the frequency of realizations with deceleration overall, showing that the phenomenon is robust to a wide range of ecological conditions (Fig. 3b). We also analysed the sensitivity of wave speed to dispersal rate (Fig. 3c,d) on a heterogeneous lattice close to the percolation threshold (*p* = 0.6). While increasing dispersal rate unsurprisingly increased wave speed, there was no threshold dispersal value below which the wave speed could not be measured, while the predicted deceleration was always present.

**Figure 4:**
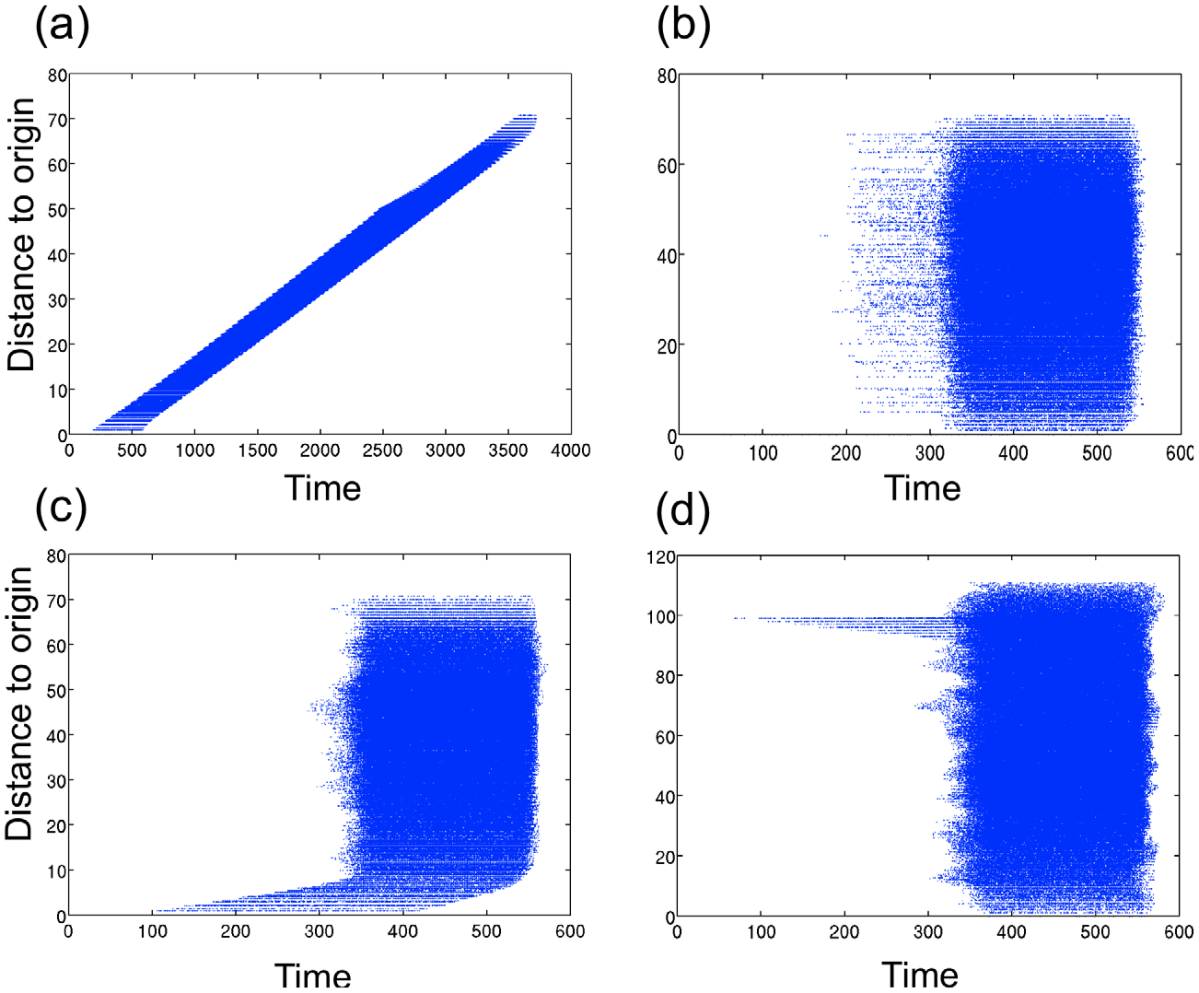
The effect of the addition of global dispersal on the displacement between infected sites and the origin with increasing time. Simulations were run on a 100×100 lattice to reproduce a spatio-temporal pattern of WNV-positive dead birds detections. At every time step, sites having more than 1% infectious reservoirs were “observed” with a probability of 0.17 to mimic under-reporting of WNV-positive dead birds. (a) With only local dispersal (1%), there is a significant positive correlation between Euclidean distance of selected sites to the origin and time (b) With only global dispersal (1%), there is no correlation between Euclidean distance to the origin and time (c) With a combination of local dispersal (0.99%) and global dispersal (0.01%), there is a significant positive correlation between the Euclidean distance and time (d) When Euclidean distance is measured from a point (false origin) that is at the opposite end of the lattice than the true origin, there is a significant negative correlation of Euclidean distance and time for the combination of local and global dispersal.

A final concern was that the preceding theoretical results were obtained under the assumption that dispersal of the pathogen was contained within the local neighbourhood of an infected site. Previous results in analogous systems have shown that the inclusion of long-distance connections reduces *p_c_* compared to exclusively local dispersal [33]. As a diagnostic for the predominance of local dispersal in the WNV data, we tested for a correlation between time elapsed since the annual index case and the Euclidean distance between each observed infection and the presumptive origin of the annual outbreak, *i.e.*, its displacement from the epicentre. Because purely local dispersal leads to a propagating wave-front, distance from the outbreak origin and time elapsed must be positively correlated. No such correlation occurs in the case of global dispersal alone, while in the mixed case the wave front only remains intact when local dispersal dominates spatial spread (Fig. 4). Applying this test to the WNV data provided strong evidence that spread of WNV was indeed dominated by local dispersal in 2000–2002 and 2004–2007, but not 2003 or 2008 (see Table S1 in Text S1). Notably, there was no evidence for dominance of local dispersal in 2008, the year in which we suspect WNV emerged at multiple locations. Finally, we simulated “mixed dispersal” scenarios in which local dispersal was combined with global dispersal, either through occasional dispersal to a random transmission-promoting site, or in the form of a small world network [34]. In these simulations, distance from the outbreak origin and time elapsed retained their positive correlation as long as local dispersal dominated and the outbreak origin was correctly identified. In conclusion, we found that decelerating travelling waves were robust to a wide range of potentially confounding factors as long as the heterogeneity in the environment was in the vicinity of the critical point.

### Percolation Conditions In Nyc

**Figure 5:**
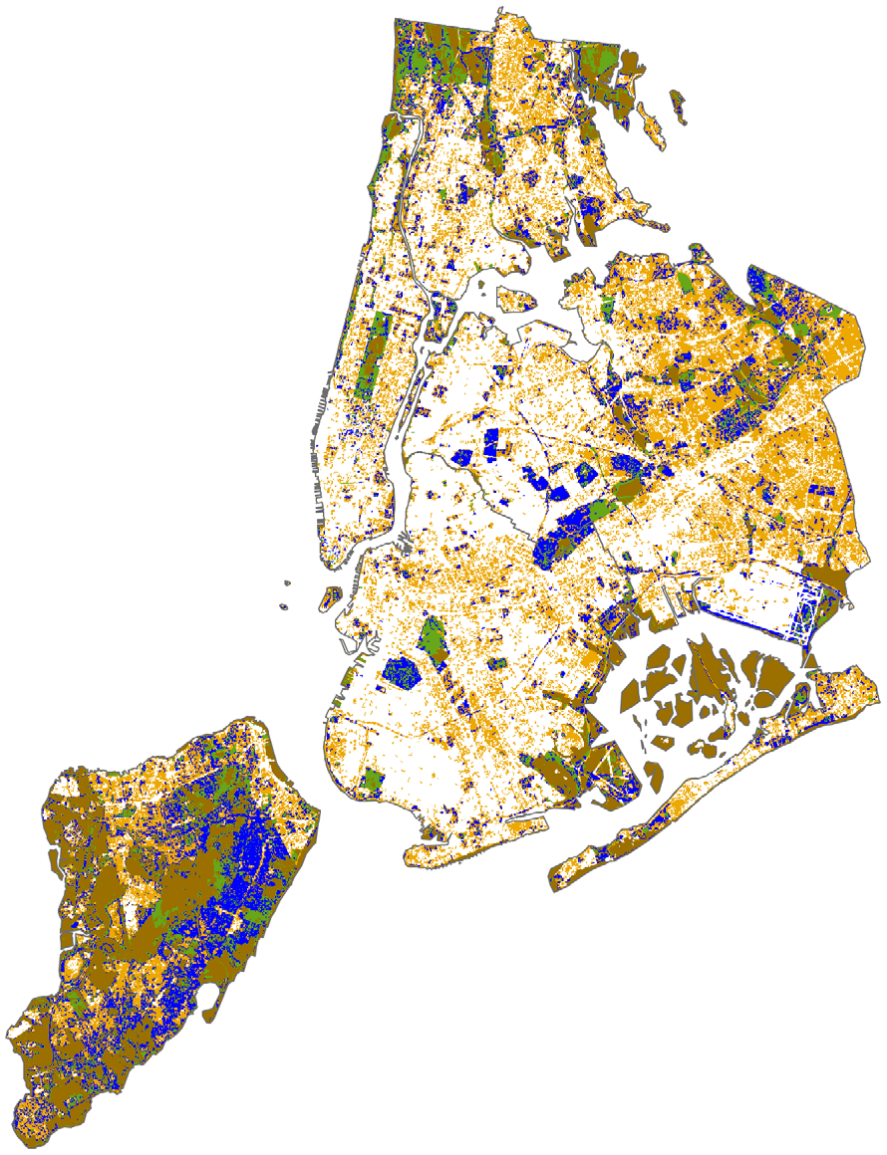
Distribution of land-cover types in NYC. White represents the unfavourable developed high-intensity land cover type. Orange, blue and green areas represent developed medium-intensity, developed low intensity, and developed open-space land cover types, respectively, while brown represent all other land-cover types. Non-white (hospitable) areas cover 59.93% of NYC.

To investigate percolation conditions in New York City, we tested for association between prevalence of WNV in birds and land cover type on a 50 m×50 m grid, the territory size of American Robin (*Turdus migratorius*), a dominant amplifying reservoir in this system ([35]; Table 2). Five land cover types (Open-Space, Low-Intensity Developed, Evergreen Forest, Herbaceous, and Woody Wetland), all of which are characterized by <50% impervious surface, were significantly associated with prevalence at the Bonferroni corrected significance level (*α*_Bonferroni_ = 0.005), suggesting that these land cover types promote the transmission of WNV. Areas of high intensity developed land cover, characterized by 80%–100% impervious surfaces and comprising 40.1% of the land surface of NYC, were significantly negatively associated with prevalence, suggesting that this land cover type is indeed an impediment to the spread of WNV in New York City. Importantly, this developed high-intensity land cover type is widely distributed throughout New York City (Fig. 5) so that transmission-promoting land cover types are scattered within a larger inhospitable matrix. Notably, the proportion of transmission-promoting land cover types was 0.599 (95% CI [0.598–0.600] from the binomial distribution), practically indistinguishable from the percolation threshold, *p*_c_ = 0.5927… for a Bernoulli site percolation on a von Neumann lattice. This agreement suggests that transmission promoting habitats in New York City are indeed in the vicinity of the critical point, though the near perfect equivalence should be interpreted with caution, since the characteristic scale of transmission and the geometry and spatial correlation of the transmission-promoting sites remain unknown. That is, our assumption of Bernoulli site percolation on a von Neumann lattice is an idealization. The idealization is justified by the biological basis of the 50 m×50 m granularity (territory size of American Robin) and indirect evidence obtained above that transmission is primarily local. To the extent that this idealization fails to capture the geometry of the environment as perceived by both vectors and hosts and/or relevant correlations, the analytic critical point (*p*_c_ = 0.5927…) only approximates the true unknown critical value. While percolation thresholds are known to vary between 0.4 and 0.8 for different site geometries, [12], the extreme values in this range are associated with rather exotic scenarios and the majority of ecologically plausible geometries give values between 0.5 and 0.6. It is therefore probable that transmission is unstable throughout the city, even if our assumptions about network geometry should prove overly simplistic.

**Table 2:** Parameters and state variables of the WNV model.

State variable/ Parameter name	Description	Value	Reference
S_R_	Susceptible reservoir		
I_R_	Infectious reservoir		
R_R_	Recovered reservoir		
N_R_	Total reservoir		
S_V_	Susceptible vectors		
E_V_	Exposed (incubating) vectors		
I_V_	Infectious vectors		
N_V_	Total vectors		
α_R_	Transmission probability vector to host	0.88	[49]–[51]
α_V_	Transmission probability host to vector	0.16	[49]–[51]
β	Biting rate	0.44/day	[52]
μ_R_	Reservoir mortality rate	0.00027/day	[53]
μ_V_	Vector mortality rate	0.03/day	[54], [55]
γ_R_	Reservoir recovery rate	0.222/day	[56] (duration of viremia 4–5 days)
δ_R_	Reservoir mortality rate due to WNV infection	0.143/day	[56]–[58]
1/κ_V_	Extrinsic incubation period in vector	9.43 days	[51]
*β_n_*	Proportion of birds dispersing between sites	0.01	

### Alternative Explanations For Decelerating Waves

An alternative explanation for the observed deceleration is that spread rate merely tracks an exogenous seasonal variable. Most plausible such possibilities were excluded by further analysis. Two candidate variables are temperature, which strongly modulates the development of the larval stage of the mosquito vector, and therefore the growth and abundance of vector populations (see Fig. 1 in [36]), and precipitation, which limits available breeding habitat for the primary vector species in NYC (*Culex pipiens*, *Cx. restuans*, and *Cx. salinarius*). Inspection of average daily temperature (obtained from reports at JFK Airport, La Guardia Airport and Central Park NOAA weather stations) and mosquito abundance overlaid on spread rate, however, show that the decline in wave speed typically precedes seasonal declines in temperature and mosquito abundance (see Fig. 1 and Figs. S1,S2 and S3 in Text S1). Further, correlations between estimated wave-speed and degree day (11°C base temperature), precipitation, total mosquito abundance and the abundance of *Culex* sp., were not statistically significant at the α = 0.05 level (using Holm-Bonferroni corrections for multiple tests), with the exception of total mosquito catch per unit effort, which was correlated with wave-speed measured in mosquitoes in 2003 using the convex hull method; and mosquito catch per unit effort for *Culex* species, which was correlated with wave-speed measured in birds and in the combined dataset in 2000, using the boundary displacement method (see Tables S2, S3 and S4 in Text S1, respectively). Given that these variables are the key determinants of vector population dynamics, it is implausible that either separately or collectively they are responsible for the decelerating spread we observed. However, we acknowledge that the pattern of decelerating wave-speed found for WNV in NYC might be explained by other alternative factors that we were unable to explore, *e.g.* the intensity of dispersal between neighbouring areas of NYC or intensity of local transmission. Our results demonstrate that spatial heterogeneity alone is sufficient to produce the decelerating pattern, and in the absence of support for alternative explanations, we propose it as the mechanism underlying the observed pattern found in New York City.

A further alternative hypothesis to explain the pattern we observed is simple stochastic fadeout, where the pathogen goes locally extinct due to a decreasing frequency of transmission events in a finite system of hosts and vectors such that the accumulation of local extinctions is manifest as a decline in spread rate. If the correct system size was known (*i.e.* the absolute rather than relative number of hosts and vectors occupying cells of the spatial model), it would be possible to investigate this hypothesis rigorously. We believe that such an analysis exceeds current capabilities, however, because choosing a sufficiently small system size will undoubtedly and artefactually lead to stochastic fadeout. Due to this ambiguity, we believe that a stochastic formulation of the model we present here would be unhelpful. In contrast, two counter-arguments suggest that the observed pattern of deceleration is unlikely the result of stochastic fadeout: (1) In all years (2000–2008) studied, WNV successfully spreads from one end of New York City (Staten Island or Queens) to the other end, whereas a stochastic fadeout would generally lead to the arrest of the pathogen in the part of the city in which it first appeared; (2) We found evidence of deceleration consistently in all years studied. If the decelerating pattern was due to stochastic fadeout, we would expect to find deceleration in a smaller subset of the annual epizootics studied, since stochastic fadeout depends by definition on random events that break the transmission chain of the pathogen. In contrast, the underlying structure of the habitat in terms of transmission-promoting and transmission-inhibiting land-cover types is constant, supporting a consistent pattern of spread. While stochastic processes undoubtedly take place during the spatial spread of WNV in NYC, we suspect that the decelerating wave pattern we found is better explained by habitat heterogeneity.

### Robust Control

**Figure 6:**
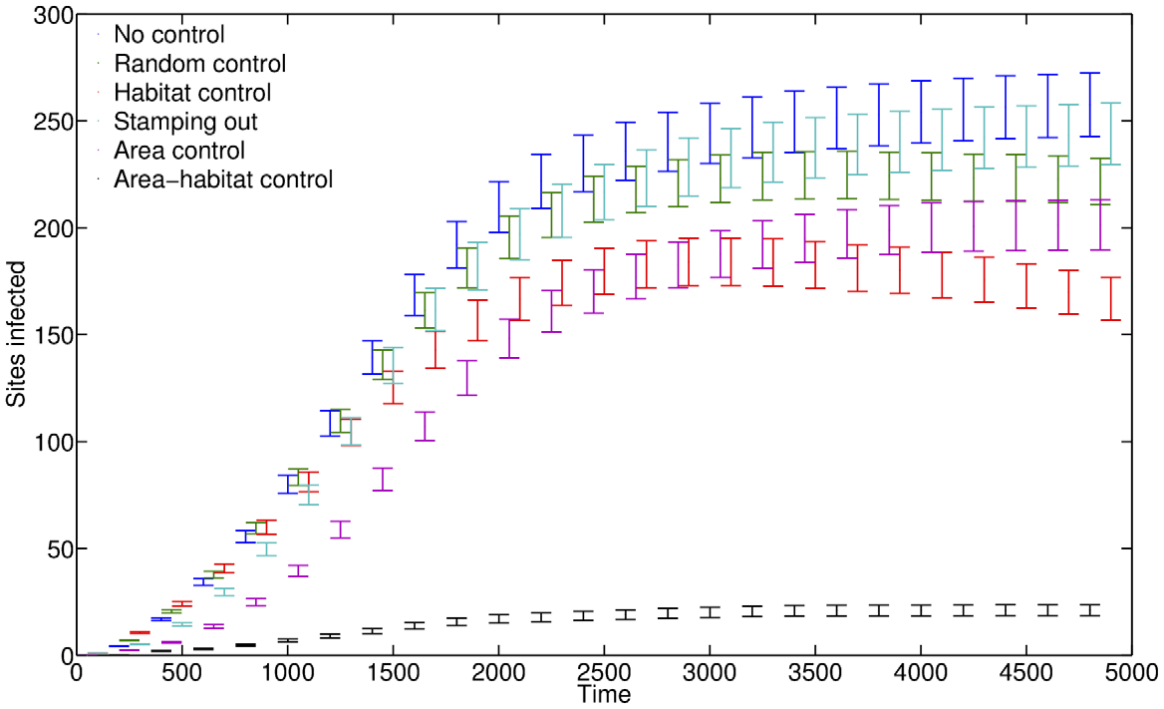
Comparison of the effectiveness of model-based and uninformed control strategies. Effectiveness is measured as the average number of sites where infectious hosts reached a threshold of 1%, with error-bars showing the standard errors (100 realizations; 30×30 heterogeneous lattice (*p* = 0.6); 5000 time-steps; dispersal rate of 0.01; vector-to-host ratio of 2). For comparison, we also depict the spread of WNV in the absence of any control for comparison (blue). All control strategies made selected sites permanently inhospitable, but differed in their mode of selection. Random control (green): random site every 25 time-steps (200 sites treated overall, 107.14±0.76 habitable sites treated); Habitat control (red): random transmission-promoting site every 25 time-steps (200 sites treated overall, all habitable); Stamping out (cyan): random site with >5% infectious hosts every time-step (16.65±1.0765 habitable sites treated overall); Area control (purple): random (Moore) neighbour of site with >5% infectious hosts every time-step (208.79±16.0279 sites treated overall, 86.84±6.6703 habitable sites treated); Area-habitat control (black): random transmission-promoting (Moore) neighbour of site with >5% infectious hosts every time-step (240.82±15.0462 sites treated overall, all habitable).

To conclude our study, we noted that this finding can be deployed to improve control. Unstable transmission implies that the spread of infection might be delayed or even halted by identifying and closing corridors of transmission that link remote susceptible areas and outbreak epicentres. Accordingly, in a final set of analyses we compared five potential control strategies according to their effectiveness in limiting the spread of WNV on a lattice with environmental heterogeneity close to the percolation threshold (Fig. 6). Of the tested strategies, the most effective was to treat transmission-promoting site locations in the immediate neighbourhood of sites at which infection exceeded a detection threshold (>5%), despite that this resulted in only modest increase in the total number of sites treated. This strategy was also the most effective when simulations were run on a lattice where all sites were transmission-promoting, although the number of sites that required treatment was considerably larger than for other control methods (Fig. S6 in Text S1). This finding that selectively blocking the propagation of WNV from highly infected sites to transmission-promoting sites in their neighbourhood is a highly effective strategy is consistent with models for other disease systems [37],[38], but has not yet been incorporated formally into vector control guidelines [39]. We hereby propose that such strategies be given consideration.

### Conclusion

Understanding the emergence and spread of vector-borne pathogens in cities remains an important problem for the ecology of infectious diseases. We have shown here that one ubiquitous property of cities, spatial heterogeneity, gives rise to endogenously decelerating waves, a phenomenon that is not known to occur elsewhere. We detected such waves in annual outbreaks of WNV in New York City between 2000 and 2008 and confirmed three important conditions for the observed deceleration to be driven by heterogeneity: (1) predominance of local dispersal, (2) association between WNV prevalence and environmental heterogeneity, in this case infection-promoting land cover types, and (3) prevalence of infection-promoting land cover types in the vicinity of the critical threshold. Our results suggest that towards the end of annual epizootics, when transmission risk to humans is the highest, the extent of the area infected is unlikely to expand considerably. To our knowledge, this is the first study to provide evidence of decelerating waves of infection due to environmental heterogeneity in the absence of a gradient, a result which supports selective treatment of transmission-promoting areas in the vicinity of infected sites as a strategy to delay or even halt disease spread.

## Materials And Methods

### Data Collection And Preparation

The data reported here were collected by the New York Department of Health and Mental Hygiene (NYCDOHMH) between 2000 and 2008. Between 2000 and 2007, dead birds were voluntarily reported by the public to the Department by phone or in person and then collected by NYCDOHMH personnel. If the condition of the carcass allowed, it was identified to species, and tested by both PCR and ELISA for live WNV as well as for antibodies against WNV. Dead birds were designated positive if both tests showed a positive response. Between 2000 and 2008, mosquitoes were collected weekly in CDC light and Reiter's gravid traps. Trap catch was separated in the lab to species, and grouped into pools of up to 50 individuals from the same species, on the same date and collected from the same trap. These pools were than tested using PCR for WNV. Geographically coded records were converted to the NAD 1983 State Plane New York Long Island FIPS 3104 coordinate system for mapping and calculation of infected area. Mapping and geostatistical analysis were performed using ESRI ArcGIS and R (ESRI ArcMap 9.2, R project [40]), using R packages PBSmapping, maptools, splancs and spatstat.

Mosquito abundance was measured as daily catch-per-unit-effort (CPUE), *i.e.*, the average number of mosquitoes collected per trap night. Because collections did not occur every day and there was substantial variation in CPUE on subsequent days, we smoothed estimated CPUE using local polynomial regression.

### Wave-Speed Estimation

We estimated the wave-speed at which WNV spread in NYC using three methods, a convex hull method, a boundary displacement method, and a maximum distance method, as recommended by [41]. The convex hull method consisted of estimating the infected area for every day during annual epizootics as the area of the convex hull encompassing all locations at which WNV was presently or previously detected and calculating daily change in the square root of this area. This method has been shown to introduce a bias if disease spread is anisotropic [31], as in our case. We corrected for this bias by measuring wave-speed as the average daily increase in the length of transects originating from the epicentre at 22.5° increments as those intersect the boundaries of the infected area on subsequent days (boundary displacement method). The maximum distance method consisted of determining the maximum displacement of locations at which WNV was detected with respect to the initial case during each annual epizootic and taking subsequent differences in this quantity. Wave-speed was estimated to be zero on days when WNV was not detected or when it was detected inside the previously estimated infected area (convex hull and boundary displacement methods) or closer to the initial case than the prior maximum extent (maximum distance method). When the infected area/distance increased, we normalized the wave-speed by dividing the calculated wave-speed by the number of days since the last observed expansion.

### Wnv Transmission Model

To model the spread of WNV in NYC we used a deterministic coupled map lattice with local dynamics given by an extended Ross-MacDonald model [42],[43] (Fig. 2a). The transmission portion of the model combines an SIR model for reservoir hosts and an SEI model for vector mosquitoes. These equations, which are derived on biological grounds, are similar, but not identical, to previously published models of WNV transmission [44]. Non-biting transmission modes of infection (host-to-host transmission through cohabitation and scavenging, as well as vector-to-vector transmission through co-feeding [45]) were initially considered, but later omitted as they affected only *R*_0_ and not the pattern of spread (Fig. S4 in Text S1). State variables and parameters are listed in Table 2. Host and vector populations were kept constant. No seasonal forcing was included to show that observed patterns of deceleration were endogenously generated by spatial heterogeneity. The transmission model is given by the following equations,and the basic reproductive number [46] was obtained using the spectral radius method [47]. To model dispersal, cells in the first order von Neumann neighbourhood were coupled by allowing a proportion (1%) of the reservoir bird population to disperse in each direction with reflecting boundary conditions at each time step (*i.e.*, site percolation). As for the analysis of land cover types, cell size is envisioned to represent the typical territory size of birds that are hosts of WNV, *i.e.*, 50 m ( 50(m, corresponding to the territory size of American Robin (*Turdus migratorius*) [35], a dominant amplifying host in this system. All rate parameters were defined in units per day. In simulations, sites were randomly assigned to transmission-promoting and uninhabitable categories with probability *p* (value depending on simulation), and uninhabitable sites were constrained to contain no mosquito or bird populations. We initialized each iteration with a single infectious host at the origin of the lattice. Wave speed of WNV in the spatial model was estimated as the rate of change in the estimated infected area encompassing all sites in which >1% of birds became infectious (100(100 lattice, 6,000 time-steps). Numerically erratic behaviour induced by the discrete lattice was smoothed by a moving average with a bandwidth of 500 days. Simulated wave-speed trend was calculated as . Final wave-speed was measured when the first site at the edge of the lattice reached 1% bird prevalence, or at the end of the simulation if the infection failed to reach an edge. We took as evidence of a decelerating wave; for visualization was averaged over 100 realizations for each set of parameters to describe the average wave-speed trend in time (Fig. 2d). In a small subset of realizations infection failed to propagate due to the lack of hospitable sites in the neighbourhood of its origin. In these cases . These realizations were nonetheless included in the calculation of average wave-speed based on the argument that many such failed attempts of spread occur in nature and are integrated into the pattern of spread for WNV in a season, such as we describe in New York City. Since one might alternatively argue that the increasing proportion of failed realizations with decreasing proportion of hospitable sites will bias the wave-speed trend, and could itself lead to an overall decelerating wave-speed close to the percolation threshold, we also calculated the conditional average wave-speed excluding failed realizations. The conditional wave-speed also showed deceleration in the vicinity of the percolation threshold (Fig. S5 in Text S1), however, in a smaller range than the unconditional wave-speed (0.54<*p*<0.58 *vs.* 0.52<*p*<0.6). We conclude that decelerating waves are not an artefact of the increasing number of failed realizations as *p* declines to the critical value.

### Effects Of Global Dispersal

To determine the sensitivity of wave speed to the assumption of local dispersal, in another set of simulations we allowed a proportion of hosts from each transmission-promoting site to disperse to a randomly chosen transmission-promoting site. In the case of global dispersal exclusively, wave speed is undefined and as the system is well-mixed. When global dispersal occurs in conjunction with local dispersal, sites that receive global dispersers initiate local spread in their vicinity if *R*_0_>1. Such long-distance connections have been shown to reduce compared to the case of exclusively local dispersal in analogous systems [33]. We also incorporated long-distance dispersal using a small world-type model, where we rewired 5% of the local connections between sites following standard methods [34]. Simulations using this model were qualitatively similar to simulations with a mixture of global and local dispersal. An alternative characteristic of local dispersal is that the Euclidean distance of the wave-front from the origin (“displacement”) increases significantly with time since the start of the outbreak. We investigated how the addition of global dispersal affects this positive correlation by measuring displacement at each time step in simulations. We labelled all sites with >1% infectious hosts in the current time step. To mimic the effect of under-reporting, each labelled site was selected with probability 0.17, the estimated reporting rate for bird decoys in urban environments [48]. Assuming strictly local dispersal, displacement indeed increased with time (Fig. 4a), while there was no correlation between displacement and time when only global dispersal was assumed (Fig. 4b). When local dispersal was supplemented by global dispersal, the positive correlation between displacement and time was retained if at least half of all dispersers spread locally (Fig. 4c). It follows that the significant positive correlation of distance to the origin and time is an indicator of the presence of an intact wave-front and therefore the dominance of local dispersal. The presence of multiple origins did not qualitatively change this pattern when distance was calculated to any of the multiple origins, as the local dispersal around any origin ensures the positive correlation. However, when distance was calculated to a putative origin that was in fact far from the true origin, distance to this false origin could be negatively correlated with time (Fig. 4d). This second criterion was therefore used to reject putative origins of the WNV epizootic in NYC.

### Wnv Prevalence Across Different Land-Cover Types In Nyc

Prevalence was estimated from the ratio of WNV-positive dead birds to all reported dead birds averaged over 2001–2007. We obtained a comprehensive land cover map for NYC using the land cover classification from the National Land Cover Dataset 2001 (http://www.mrlc.gov/nlcd_multizone_map.php). We assigned each recovered bird carcass to the unique land cover type in which it was found and performed pair-wise χ^2^ tests on the number of WNV-positive and total dead birds found in each land-cover type versus all other land-cover types to test the hypothesis of homogeneity (Table 3). There is strong evidence in the literature that detection and reporting rates of birds differ across land-cover types [48]. However, there is no evidence that the detection and reporting rates of WNV-positive and negative dead birds is significantly different. Since we estimate the WNV prevalence across land-cover types by the ratio of WNV-positive to all dead birds reported, we assume only that the detection and reporting rates of WNV-positive and negative dead birds are the same. In this case, differences in detection and reporting rates of both WNV-positive and all dead birds across land-cover types cancel out in the calculation of WNV prevalence.

**Table 3:** Heterogeneity of WNV-prevalence across different land-cover types in NYC.

Land-cover type	Area (%)	WNV prevalence	Expected WNV prevalence	κ^2^	*p*
Developed, open space	6.15	34	18.24	**14.03**	**<0.001**
Developed, low intensity	11.9	107	80.94	**9.65**	**0.002**
Developed, medium intensity	28.14	262	246.52	1.62	0.2
Developed, high intensity	40.07	187	263.79	**38.96**	**<0.001**
Barren land	0.63	1	0.21	2.87	0.09
Deciduous forest	3.72	2	2.81	0.23	0.628
Evergreen forest	0.54	7	0.43	**100.56**	**<0.001**
Mixed forest	0.11	2	0.61	3.18	0.074
Crops	0.84	1	0.48	0.55	0.457
Woody wetland	1.99	10	3.06	**15.81**	**<0.001**
Herbaceous wetland	2.68	6	1.9	**8.9**	**0.003**

Degrees of freedom was 1, and a Bonferroni-corrected significance level of α = 0.005 was used. Table only lists land-cover types in which WNV-positive dead birds were found. Land-cover types significantly different from the average are bold.

## Supporting Information

Text S1 Supplementary information. (PDF)
