# Supplementary material for: Decelerating Spread of West Nile Virus by Percolation in a Heterogeneous Urban Landscape
Source: PLoS Comput Biol. 2011 Jul 28;7(7):e1002104. doi: 10.1371/journal.pcbi.1002104 (PMC3145642; doi:10.1371/journal.pcbi.1002104)
Supplement: Text S1 — (PDF) [file pcbi.1002104.s001.pdf]

Decelerating spread of West Nile virus due to percolation in a heterogeneous, urban landscape

Text S1

Krisztian Magori[1], Waheed I Bajwa[2], Sarah Bowden[1] and John M. Drake[1]
[1] *Odum School of Ecology, University of Georgia, Athens, GA 30602*
[2] *Office of Vector Surveillance and Control, New York City Department of Health and Mental Hygiene, New York, NY 10013*

1 Stability analysis of the local WNV transmission model

The basic reproductive ratio [1], R_0, of the local WNV model (Eq. 2-6), obtained using the spectral radius method [2], may be expressed as

$$R_0 = \sqrt{\alpha_V \alpha_R \beta^2 \frac{N_V}{N_R} \frac{\kappa_V}{\kappa_V + \mu_V} \frac{1}{\mu_V(\delta_R + \mu_R + \gamma_R)}}. \tag{S1}$$

This expression is the square root of the next generation reproduction number which assumes that the pathogen must pass through both the vector and the host to be counted a novel infection. Note that the two expressions agree on the critical point $R_0 = 1$ since $\sqrt{1} = 1$. The next generation reproduction number may be derived as follows. In order for WNV to spread in the host-vector system, it has to be able to spread from a host to a vector, and from a vector to a host, independently and simultaneously. Thus, the system has to satisfy the following simultaneous per capita positive growths in the host and vector subsystems:

$$\lim_{I_R \to 0} \left(\frac{1}{I_R} \frac{dI_R}{dt} \right) > 0, \quad \lim_{E_V, I_V \to 0} \left(\frac{1}{E_V} \frac{dE_V}{dt}, \frac{1}{I_V} \frac{dI_V}{dt} \right) > 0. \tag{S2}$$

Based on Eq. (3), the first condition is satisfied if

$$I_R/I_V < \frac{\alpha_R \beta}{(\mu_R + \delta_R + \gamma_R)}. \tag{S3}$$

Based on Eqs. (6)-(7), the second condition is satisfied if

$$I_R/I_V > \frac{\mu_V(\mu_V + \kappa_V)}{\kappa_V \alpha_V \beta} \frac{N_R}{N_V}. \tag{S4}$$

Combining (A2) and (A3) and rearranging terms gives

$$R_0 \equiv \frac{\alpha_R \alpha_V \kappa_V \beta^2}{\mu_V(\mu_V + \kappa_V)(\mu_R + \delta_R + \gamma_R)} \left(\frac{N_V}{N_R} \right) > 1. \tag{S5}$$

When $\frac{N_V}{N_R} < \frac{\mu_V(\kappa_V+\mu_V)(\delta_R+\gamma_R+\mu_R)}{\alpha_R \alpha_V \beta^2 \kappa_V}$ (0.7184, using parameters from Table 2) this system only admits the disease free equilibrium (DFE) ($S_R^* = N_R, I_R^* = 0, R_R^* = 0, S_V^* = N_V, E_V^* = 0, I_V^* = 0$). When $\frac{N_V}{N_R} > \frac{\mu_V(\kappa_V+\mu_V)(\delta_R+\gamma_R+\mu_R)}{\alpha_R \alpha_V \beta^2 \kappa_V}$, the equilibrium solutions are the DFE and an endemic equilibrium at:

$$S_R^* = \frac{\mu_R {N_R}^2(\gamma_R + \delta_R + \mu_R)[{\mu_R}^2 {N_R}^2 + 2\mu_R N_R \alpha_R \beta I_V]}{{\mu_R}^3 {N_R}^3(\gamma_R + \delta_R + \mu_R) + 2{\mu_R}^2 {N_R}^2 \alpha_R \beta I_V(1.5\mu_R + 1.5\gamma_R + \delta_R) + \mu_R N_R(\alpha_R \beta I_V)^2(3\gamma_R + 3\mu_R + \delta_R) + (\gamma_R + \mu_R)(\alpha_R \beta I_V)^3}. \tag{S6}$$

$$I_R^* = \frac{\alpha_R \beta I_V \mu_R N_R}{(\gamma_R + \delta_R + \mu_R)\mu_R N_R + \alpha_R \beta I_V (\gamma_R + \mu_R)}. \tag{S7}$$

$$R_R^* = \frac{\alpha_R \beta I_V \gamma_R N_R}{(\gamma_R + \delta_R + \mu_R)\mu_R N_R + \alpha_R \beta I_V (\gamma_R + \mu_R)}. \tag{S8}$$

$$S_V^* = \frac{\mu_V N_V N_R}{\mu_V N_R + \alpha_V \beta I_R}. \tag{S9}$$

$$E_V^* = \frac{\alpha_V \beta I_R \mu_V N_V}{(\mu_V + \kappa_V)(\mu_V N_R + \alpha_V \beta I_R)}. \tag{S10}$$

$$I_V^* = \frac{\alpha_V \beta I_R \kappa_V N_V}{(\mu_V + \kappa_V)(\mu_V N_R + \alpha_V \beta I_R)}. \tag{S11}$$

In the absence of infectious vectors, the formula for susceptible hosts simplifies to $S_R = N_R$, while I_R and R_R becomes 0. In the absence of infectious hosts, the formula for susceptible vectors simplifies to $S_V = N_V$, while E_V and I_V become 0. Except for the formula for susceptible hosts, all other equilibrium solutions are fairly simple and contain terms that have biological relevance (e.g., $(\mu_V + \kappa_V)$ and $(\gamma_R + \mu_R + \delta_R)$ are the rates at which exposed vectors and infectious hosts are removed from the population, respectively). By substituting I_V^* into Eqns. (2)-(4), and I_R^* into Eqns. (5)-(7), we obtained the following closed form expressions.

$$S_R^* = \frac{{N_R}^2(\gamma_R + \delta_R + \mu_R)(\mu_V + \kappa_V)(\alpha_V \beta \mu_R + \mu_R \mu_V + \gamma_R \mu_V)}{\alpha_R \alpha_V \beta^2 \kappa_V N_V (\gamma_R + \mu_R) + \alpha_V \beta \mu_R N_R (\gamma_R + \delta_R + \mu_R)(\mu_V + \kappa_V)}. \tag{S12}$$

$$I_R^* = \frac{\alpha_R \alpha_V \beta^2 \mu_R \kappa_V N_R N_V - \mu_R \mu_V N_R^2 (\gamma_R + \delta_R + \mu_R)(\mu_V + \kappa_V)}{\alpha_R \alpha_V \beta^2 \kappa_V N_V (\gamma_R + \mu_R) + \alpha_V \beta \mu_R N_R (\gamma_R + \delta_R + \mu_R)(\mu_V + \kappa_V)}. \tag{S13}$$

$$R_R^* = \frac{\alpha_R \alpha_V \beta^2 \gamma_R \kappa_V N_R N_V - \gamma_R \mu_V N_R^2 (\gamma_R + \delta_R + \mu_R)(\mu_V + \kappa_V)}{\alpha_R \alpha_V \beta^2 \kappa_V N_V (\gamma_R + \mu_R) + \alpha_V \beta \mu_R N_R (\gamma_R + \delta_R + \mu_R)(\mu_V + \kappa_V)}. \tag{S14}$$

$$S_V^* = \frac{\alpha_R \beta \kappa_V \mu_V N_V (\gamma_R + \mu_R) + \mu_V \mu_R N_R (\gamma_R + \delta_R + \mu_R)(\mu_V + \kappa_V)}{\alpha_R \beta \kappa_V \mu_V (\gamma_R + \mu_R) + \alpha_R \alpha_V \beta^2 \kappa_V \mu_R}. \tag{S15}$$

$$E_V^* = \frac{\alpha_R \alpha_V \beta^2 \kappa_V \mu_V \mu_R N_V - \mu_V^2 \mu_R N_R (\gamma_R + \delta_R + \mu_R)(\mu_V + \kappa_V)}{\alpha_R \alpha_V \beta^2 \kappa_V \mu_R (\mu_V + \kappa_V) + \alpha_R \beta \kappa_V \mu_V (\mu_V + \kappa_V)(\gamma_R + \mu_R)}. \tag{S16}$$

$$I_V^* = \frac{\alpha_R \alpha_V \beta^2 \kappa_V \mu_R N_V - \mu_V \mu_R N_R (\gamma_R + \delta_R + \mu_R)(\mu_V + \kappa_V)}{\alpha_R \alpha_V \beta^2 \mu_R (\mu_V + \kappa_V) + \alpha_R \beta \mu_V (\mu_V + \kappa_V)(\gamma_R + \mu_R)}. \tag{S17}$$

We analyzed the stability of both the disease free equilibrium and the endemic equilibrium by studying the eigenvalues of the linearization around these steady states. All eigenvalues of the Jacobian matrix of Eqns. (2-7) must have negative real part for the steady state to be asymptotically stable. The eigenvalues themselves cannot be obtained symbolically due to the dimensionality of the characteristic equation. However, we can use Descartes's rule of signs to show that all coefficients (a_0 to a_6) have to be positive for all eigenvalues to have negative real parts. The full Jacobian matrix is

$$J = \begin{bmatrix} -\mu_R - \frac{\alpha_R \beta I_V^*}{N_R} & \alpha_R \beta \frac{I_V^*}{N_R} & 0 & 0 & 0 & 0 \\ \delta_R & -(\delta_R + \gamma_R + \mu_R) & \gamma_R & -\frac{\alpha_V \beta S_V^*}{N_R} & \frac{\alpha_V \beta S_V^*}{N_R} & 0 \\ 0 & 0 & -\mu_R & 0 & 0 & 0 \\ 0 & 0 & 0 & -\mu_V - \frac{\alpha_V \beta I_R^*}{N_R} & \frac{\alpha_V \beta I_R^*}{N_R} & 0 \\ 0 & 0 & 0 & 0 & -(\kappa_V + \mu_V) & \kappa_V \\ -\frac{\alpha_R \beta S_R^*}{N_R} & \frac{\alpha_R \beta S_R^*}{N_R} & 0 & 0 & 0 & -\mu_V \end{bmatrix} \quad \text{(S18)}$$

At the DFE, this simplifies to:

$$J = \begin{bmatrix} -\mu_R & 0 & 0 & 0 & 0 & 0 \\ \delta_R & -(\delta_R + \gamma_R + \mu_R) & \gamma_R & -\alpha_V \beta \frac{N_V}{N_R} & \alpha_V \beta \frac{N_V}{N_R} & 0 \\ 0 & 0 & -\mu_R & 0 & 0 & 0 \\ 0 & 0 & 0 & -\mu_V & 0 & 0 \\ 0 & 0 & 0 & 0 & -(\kappa_V + \mu_V) & \kappa_V \\ -\alpha_R \beta & \alpha_R \beta & 0 & 0 & 0 & -\mu_V \end{bmatrix} \quad \text{(S19)}$$

The three coefficients corresponding to the three highest order terms of the characteristic equation of this Jacobian (a_0 to a_2) are positive definite. Given that all parameters of the model are positive, the signs of the remaining coefficients depend on the ratio of vectors to reservoirs. The constant coefficient a_6 is positive if and only if the inequality

$$\frac{N_V}{N_R} < \frac{\mu_V(\kappa_V + \mu_V)(\delta_R + \gamma_R + \mu_R)}{\alpha_R \alpha_V \beta^2 \kappa_V}. \quad \text{(S20)}$$

holds, which exactly corresponds to $R_0 < 1$ (see Eq. 1). The solution of the equation $a_6 = 0$ for N_V gives the critical ratio of vectors to reservoirs for $R_0 = 1$ in terms of model parameters. We also expressed the critical N_V for $a_3 = 0$, $a_4 = 0$ and $a_5 = 0$, respectively. The difference between the critical N_V for $a_6 = 0$ and for the other three coefficients were all positive, as they contained only positive terms. Therefore, we conclude that inequality (S20) is a necessary and sufficient condition for the disease-free equilibrium to be asymptotically stable.

In the case of the endemic equilibrium, a similar analysis shows that the constant coefficient a_6 of the characteristic equation of the Jacobian evaluated at the endemic equilibrium is positive if and only if:

$$\frac{N_V}{N_R} > \frac{\mu_V(\kappa_V + \mu_V)(\delta_R + \gamma_R + \mu_R)}{\alpha_R \alpha_V \beta^2 \kappa_V}. \tag{S21}$$

which corresponds to $R_0 > 1$. However, there are two sets of solutions for the critical N_V for $a_3 = 0$, $a_4 = 0$ and $a_5 = 0$, which only differ in the sign of a square root term. We were able to show that the difference between the critical N_V for $a_6 = 0$ and for the other three coefficients were all positive for the set of those solutions with the positive square root term. However, we weren't able to show the same for the set of solutions with the negative square root term. Evaluating all solutions for the critical N_V at the default parameter values used in Table 1 showed that indeed the N_V corresponding to inequality (S21) is the largest. Therefore, we concluded that inequality (S21) is a necessary and sufficient condition for the endemic equilibrium to be asymptotically stable.

2 Presumed origins for annual WNV epizootics in NYC

To use the significantly positive correlation of the distance from the origin and time to detect the dominance of local dispersal in our data, we located the presumptive origin of the WNV epizootic in NYC for every year studied. However, unlike in our spatial simulations, it was impossible to definitively identify the true origin of each annual epizootic due to under-reporting of dead birds and the uneven distribution of mosquito surveillance locations. Therefore, we aimed to identify the presumptive origin of the WNV epizootic in NYC for each year between 2000-2008 based on the combination of WNV-positive mosquito pools tested and dead birds reported (collectively cases). For each year, we identified two putative origins based on the reported location and date of all reported cases. The location and date of the first case was always included as a putative origin. The second putative origin was assigned to the location and date of the case that had the maximum ratio of Euclidean distance and date difference to the first case, excluding cases that were less than 30,000 feet away from the first case to rule out common origin. For each case, we calculated the Euclidean distance to, and time elapsed since, each putative origin. We separated cases into two clusters (cluster 1 and cluster 2) based on which of the two putative origins each was closest to, irrespective of the number of days between the dates of reporting. Using a Spearman rank-order correlation, we tested for correlation between the Euclidean distance of the members of these clusters to their respective putative origin and the difference of the dates when they were reported to the date of the putative origin. We interpreted a significant positive correlation as an indication that the putative origin was close to the true origin of the (annual) epizootic. We interpreted a significant negative correlation as an indication that the putative origin was far from the true origin. This procedure was repeated under the assumption of a single putative origin and a single combined cluster of all cases. Throughout, we adopted a significance level of $\alpha = 0.05$.

The findings of this analysis are summarized in Table S1. In 8 out of 9 years, we were able to identify a putative origin with significant positive correlation between Euclidean distance and differences in dates for all cases. This supports the argument that, at least in these years, there was a single origin of the WNV epizootic in New York City.

2000

The first WNV-positive dead bird in 2000 was reported in Queens on July 3rd (day 184). The first WNV-positive mosquito pool in 2000 was found in Staten Island on July 7th (day 188). Three additional WNV-positive dead birds were reported on July 5th (day 186) in three different locations, of which the furthest from the first reported WNV-positive dead bird was found in Staten Island. The location and date of this WNV-positive dead bird was selected as the second putative origin by the criteria detailed above. There was no evidence for a correlation between Euclidean distance and time difference for the first putative presumed origin (Queens) with only cluster 1 included. With all cases included, there was a significant negative correlation between Euclidean distance and time to the first putative origin. However, there was significant positive correlation between the Euclidean distance to, and the time since the second putative origin (Staten Island), including only cluster 2 as well as including all cases reported. Therefore, we selected the location and date of the second putative origin in Staten Island as the presumed origin of the WNV epizootic in NYC for 2000.

2001

The first WNV-positive dead bird was reported in 2001 on day June 29th (day 179) in the Bronx. The first WNV-positive mosquito pool was found on July 3rd (day 183) in Queens. An additional WNV-positive dead bird was reported on July 5th (day 185) on Staten Island. Two additional mosquito pools tested positive on July 6th (day 186) on Staten Island. The location and date of the second WNV-positive dead bird was selected as the second putative origin. We found significant negative correlation between the distance to and the time since the first putative origin (Bronx), both with only cluster 1 and with all cases included. There was a significant positive correlation between the Euclidean distance to and the time since the second putative (Staten Island) origin, both with only cluster 2 or all cases included. Therefore, we selected the location and date of the second putative origin in Staten Island as the presumptive origin of the WNV epizootic in NYC based on WNV-positive mosquitoes for 2001.

2002

The first mosquito pool tested positive in 2002 on June 25th (day 175) on Staten Island. The first WNV-positive dead bird was reported on June 26th (day 176) also on Staten Island. A WNV-positive mosquito pool collected on July 11th (day 191) in the Bronx was selected as the second putative origin. There was

significant positive correlation between the Euclidean distance to and the time since the first putative origin for both cluster 1 and all cases. However, we found no evidence for a correlation at the $\alpha = 0.05$ level between the Euclidean distance to and the time since the second putative origin with only cluster 2 included, and a significant negative correlation for all cases. Therefore, we selected the first putative origin (Staten Island) as the presumptive origin for the WNV epizootic in NYC for 2002.

2003

The first WNV-positive mosquito pool was collected in 2003 on July 15th (day 195) on Staten Island. Two additional WNV-positive mosquito pool was collected on July 17th (day 197) in Queens. The first WNV-positive dead bird was also reported on July 17th (day 197) in Queens. The furthest of the two WNV-positive mosquito pools found in Queens was selected as the second putative origin. We found a significantly positive correlation of distance and time for the first putative origin (Staten Island) with only including cluster 1, but no evidence for correlation when all cases were included. There was no evidence for a correlation between distance and time for the second putative origin (Queens) with either only cluster 2 or all cases included. Therefore, we selected the first putative origin on Staten Island as the presumptive origin of the WNV-epizootic in NYC in 2003.

2004

The first WNV-positive mosquito pool was collected in 2004 on June 23rd (day 174) on Staten Island. The first WNV-positive dead bird was reported on July 15th (day 196) on Staten Island. We selected the location and date of a WNV-positive dead bird reported on July 27th (day 208) in Bronx as the second putative origin. There was no evidence for a correlation between the distance to and time since their respective putative origins for both cluster 1 and cluster 2 analyzed separately. However, we found a significant positive correlation between distance to and time since the first putative origin (Staten Island) with all cases included. We also found a significantly negative correlation between distance to and time since the second putative origin (Bronx) with all cases included. Therefore, we selected the first putative origin (Staten Island) as the presumptive origin for the WNV-epizootic in NYC for 2004.

2005

Two WNV-positive dead birds were reported in 2005 on July 1st in Queens. WNV was next detected in the first WNV-positive mosquito pool on July 19th (day 199) in Bronx. We selected the date and location of the first WNV-positive mosquito pool as the second putative origin. We found a significant positive correlation between the distance to and the time since the first putative origin (Queens) with only cluster 1 included, but no evidence for a correlation when all

cases were studied. There was a significant positive correlation between distance to and time since the second putative origin (Bronx) both with only cluster 2 and all cases included. Therefore, we selected the second putative origin in Bronx as the presumptive origin of the WNV-epizootic in NYC in 2004.

2006

The first WNV-positive mosquito pool was collected in 2006 on June 27th (day 177) on Staten Island. The first WNV-positive dead bird was only reported on July 12th (day 192) on Staten Island. We selected the location and date of a WNV-positive mosquito pool collected on July 6th (day 186) in Queens as the second putative origin. We found a significantly negative correlation between distance to and time since the first putative origin (Staten Island) with only cluster 1 included and no significant correlation between distance to and time since the second putative origin (Queens) with only cluster 2 included. However, there was a significantly positive correlation between distance to and time since the first putative origin (Staten Island) with all cases included. We also found a significant negative correlation between distance to and time since the second putative origin (Queens) with all cases included. Therefore, we selected the first putative origin in Staten Island as the presumptive origin of the WNV-epizootic in NYC in 2006.

2007

The first mosquito pool tested WNV-positive in 2007 on July 18th (day 198) in Queens. The first WNV-positive dead bird was reported on the same day on Staten Island. We selected the locations and dates of these two cases as the putative origins. We found a significant positive correlation between distance to and time since the first putative origin (Queens) both with only cluster 1 as well as all cases included. However, there was no evidence for a correlation between distance to and time since the second putative origin (Staten Island) either with only cluster 2 or all WNV-positive mosquito pools included. Therefore, we selected the first putative origin (Queens) as the presumptive origin of the WNV-epizootic in NYC in 2007.

2008

The first WNV-positive mosquito pool was collected in 2008 on June 13th (day 164) on Staten Island. An additional WNV-positive mosquito pool was found on June 26th (day 177) in Queens. Data collection through dead bird reporting was stopped starting 2008. We selected the locations and dates of these two WNV-positive mosquito pools as putative origins. There was no evidence for a correlation between the distance to and the time since the first putative origin (Staten Island) with only cluster 1 as well as all WNV-positive mosquito pools included. However, we found a significant positive correlation between the distance to and time since the second putative origin (Queens) with only cluster 2

included but not when all WNV-positive mosquito pools were included. Therefore, we selected the second putative origin in Queens as the presumptive origin of the WNV-epizootic in NYC in 2008.

3 Supplementary References

1. Anderson RM, May RM (1992) Infectious Diseases of Humans. Oxford: Oxford University Press

2. van den Driessche P, Watmough J (2002) Reproduction numbers and subthreshold endemic equilibria for compartmental models of disease transmission. Math. Biosci. 180: 29-48.

Table S 1: Evidence for the dominance of local dispersal of WNV in NYC for 2000-2008 based on two-tailed Spearman rank correlation of the Euclidean distance and number of days elapsed since the presumptive index case.

	Cluster 1		Cluster 2		Cluster 1(2)		Cluster 2(1)	
Year	ρ	p-value	ρ	p-value	ρ	p-value	ρ	p-value
2000	0.073	0.403	0.293	<0.001	-0.335	< 0.001	*0.326*	*<0.001*
2001	0.179	0.038	0.364	<0.001	-0.18	<0.001	*0.279*	*<0.001*
2002	0.465	<0.001	0.021	0.779	*0.258*	*<0.001*	-0.234	<0.001
2003	0.307	<0.001	0.04	0.494	*0.053*	*0.266*	-0.061	0.2
2004	0.011	0.888	0.078	0.586	*0.145*	***0.03***	-0.236	<0.001
2005	0.343	<0.001	0.31	0.029	0.057	0.474	*0.541*	*<0.001*
2006	-0.209	0.003	0.034	0.808	*0.203*	*0.001*	-0.552	<0.001
2007	0.375	<0.001	0.186	0.2015	*0.408*	*<0.001*	-0.124	0.076
2008	0.013	0.913	0.324	< 0.001	0.01	0.888	*0.032*	*0.658*

Values in red are significant at the Holm-Bonferroni level, while bold figures are significant at the $\alpha = 0.05$ level. Numbers in italics correspond to the putative origin that we selected as the presumptive origin. Column Cluster 1 denotes the correlation for mosquitoes and dead birds that were found/collected closer to the first putative origin than to the second putative origin for each year. Column Cluster 2 denotes the correlation for the remaining subset of cases. Column Cluster 1(2) describes the correlation for all WNV-positive mosquitoes and dead birds reported/collected with regards to the first putative origin. Column Cluster 2(1) shows the same by assuming the second putative origin as the common origin.

Table S 2: Significance (p-values) of one-tailed Spearman rank-order correlation between wave-speed measured using the convex hull method in birds, mosquitoes and both, and a set of alternative explanatory variables in the study period 2000-2008.

	Year	Degree Day	Total CPUE	Culex CPUE	Smoothed Total CPUE	Smoothed Culex CPUE	Precipitation
Birds	2000	1.0000	0.5733	0.4554	0.5754	**0.0173**	0.5334
	2001	0.9394	0.4433	0.5771	0.8586	0.8586	0.9463
	2002	0.9987	0.4738	0.7321	0.8533	0.9051	0.5647
	2003	1.0000	0.7561	0.8222	1.0000	1.0000	0.6105
	2004	0.2550	0.0784	0.2177	0.9656	0.8689	0.0433
	2005	0.8533	0.8930	0.7906	0.5136	0.5136	**0.0118**
	2006	0.9998	0.9648	0.8017	0.9959	0.9940	0.3254
	2007	0.2772	0.6204	0.7403	0.9790	0.9912	0.9874
Mosquito	2000	0.8623	0.6455	0.2378	0.4447	0.9453	0.9108
	2001	0.9998	0.9873	0.9795	0.9875	0.9875	0.8980
	2002	0.9985	0.9209	0.6598	0.5790	0.6669	0.7755
	2003	0.9985	0.9874	0.9722	**0.0002**	0.9985	0.8576
	2004	0.9788	0.9286	0.9943	0.9994	0.9995	0.6110
	2005	0.9407	0.9911	0.9928	0.9983	0.9974	0.1584
	2006	0.9994	0.9626	0.9224	0.9674	0.9900	0.1501
	2007	0.8841	0.9999	0.9984	0.9999	0.9999	0.7533
	2008	0.9999	0.9190	0.9216	0.9287	0.9287	0.0746
Combined	2000	1.0000	0.8091	0.6044	0.8297	**0.0183**	0.8041
	2001	0.9980	0.9549	0.9651	0.9960	0.9960	0.9754
	2002	1.0000	0.8624	0.7889	0.9749	0.9851	0.7842
	2003	1.0000	0.9933	0.9923	1.0000	1.0000	0.7376
	2004	0.8116	0.6934	0.9156	0.9999	0.9992	0.2935
	2005	0.9981	0.9820	0.9715	0.8973	0.8973	**0.0269**
	2006	1.0000	0.9391	0.8150	0.9916	0.9981	0.0924
	2007	0.9512	0.9997	0.9935	1.0000	1.0000	0.8266

Bold values are significant at the $\alpha = 0.05$ level, while the red value is significant at the Bonferroni corrected level.

Table S 3: Significance (p-values) of one-tailed Spearman rank-order correlation between wave-speed measured using the maximum distance method in birds, mosquitoes and both, and a set of alternative explanatory variables in the study period 2000-2008.

	Year	Degree Day	Total CPUE	Culex CPUE	Smoothed Total CPUE	Smoothed Culex CPUE	Precipitation
Birds	2000	0.967	0.573	0.367	**0.009881**	0.265	0.435
	2001	0.861	0.987	0.719	0.956	0.940	0.349
	2002	0.914	0.927	0.807	0.942	0.952	0.169
	2003	0.966	0.958	0.983	0.980	0.995	0.924
	2004	0.689	0.816	0.858	0.960	0.942	0.395
	2005	0.811	0.804	0.866	0.834	0.880	**0.029**
	2006	0.988	0.996	0.867	0.990	0.991	0.421
	2007	0.933	0.964	0.412	0.995	0.981	0.227
Mosquito	2000	0.967	0.783	0.455	0.131	0.552	0.214
	2001	0.687	0.995	0.996	0.995	0.995	0.719
	2002	0.689	0.340	0.456	0.978	0.978	0.658
	2003	0.761	0.500	0.486	**0.00988**	1.000	0.761
	2004	0.992	0.901	0.980	0.993	0.989	0.865
	2005	0.820	0.425	0.110	0.719	0.615	0.975
	2006	0.904	0.837	0.707	0.870	0.928	0.918
	2007	0.126	0.886	0.762	0.736	0.897	0.592
	2008	0.950	0.748	0.800	0.978	0.965	0.179
Combined	2000	0.953	0.076	0.648	**0.00712**	0.253	0.611
	2001	0.268	1.000	0.982	1.000	0.994	0.932
	2002	0.843	0.740	0.812	0.995	0.995	0.596
	2003	0.872	0.507	0.494	0.830	0.939	0.940
	2004	0.986	0.914	0.982	0.998	0.994	0.792
	2005	0.930	0.253	0.218	0.816	0.848	0.964
	2006	0.904	0.837	0.707	0.870	0.928	0.918
	2007	0.318	0.719	0.793	0.641	0.762	0.131

Bold values are significant at the $\alpha = 0.05$ level.

Table S 4: Significance (p-values) of one-tailed Spearman rank-order correlation between wave-speed measured using the boundary displacement method in birds, mosquitoes and both, and a set of alternative explanatory variables in the study period 2000-2008.

	Year	Degree Day	Total CPUE	Culex CPUE	Smoothed Total CPUE	Smoothed Culex CPUE	Precipitation
Birds	2000	1.000	0.4235	0.192	0.281	8.5310^{-5}	0.676
	2001	0.974	0.849	0.803	0.998	0.999	0.703
	2002	0.999	0.921	0.933	0.949	0.9845	0.242
	2003	0.999	0.998	0.9985	1.000	1.000	0.489
	2004	0.692	0.688	0.902	0.927	0.835	0.624
	2005	0.851	0.773	0.9095	0.956	0.956	0.135
	2006	0.988	0.991	0.556	0.9900	0.889	0.218
	2007	0.733	0.548	0.688	0.919	0.974	0.543
Mosquitoes	2000	0.5865	0.854	0.961	**0.0117**	0.513	0.564
	2001	0.998	0.985	0.997	0.999	1.000	0.870
	2002	0.999	0.5535	0.517	0.323	0.7873	0.731
	2003	0.999	0.990	0.998	**0.0011**	1.000	0.998
	2004	0.984	0.9575	0.989	0.998	0.993	0.954
	2005	0.998	0.997	0.990	0.995	0.998	0.805
	2006	0.998	0.760	0.823	0.973	0.990	0.317
	2007	0.988	0.9995	0.999	1.000	1.000	0.143
	2008	1.000	0.881	0.936	0.992	0.995	0.400
Combined	2000	1.000	0.504	0.214	0.25	2.4410^{-5}	0.702
	2001	1.000	0.991	0.9895	0.980	0.976	0.8444
	2002	1.000	0.859	0.788	0.963	0.999	0.607
	2003	1.000	0.988	0.997	1.000	1.000	0.340
	2004	0.954	0.965	0.994	0.999	0.995	0.931
	2005	0.994	0.978	0.988	0.994	0.996	0.331
	2006	1.000	0.984	0.919	0.994	0.998	0.158
	2007	1.000	1.000	0.999	1.000	1.000	0.067

Bold values are significant at the $\alpha = 0.05$ level, while values in red are significant at the Holm-Bonferroni corrected level.

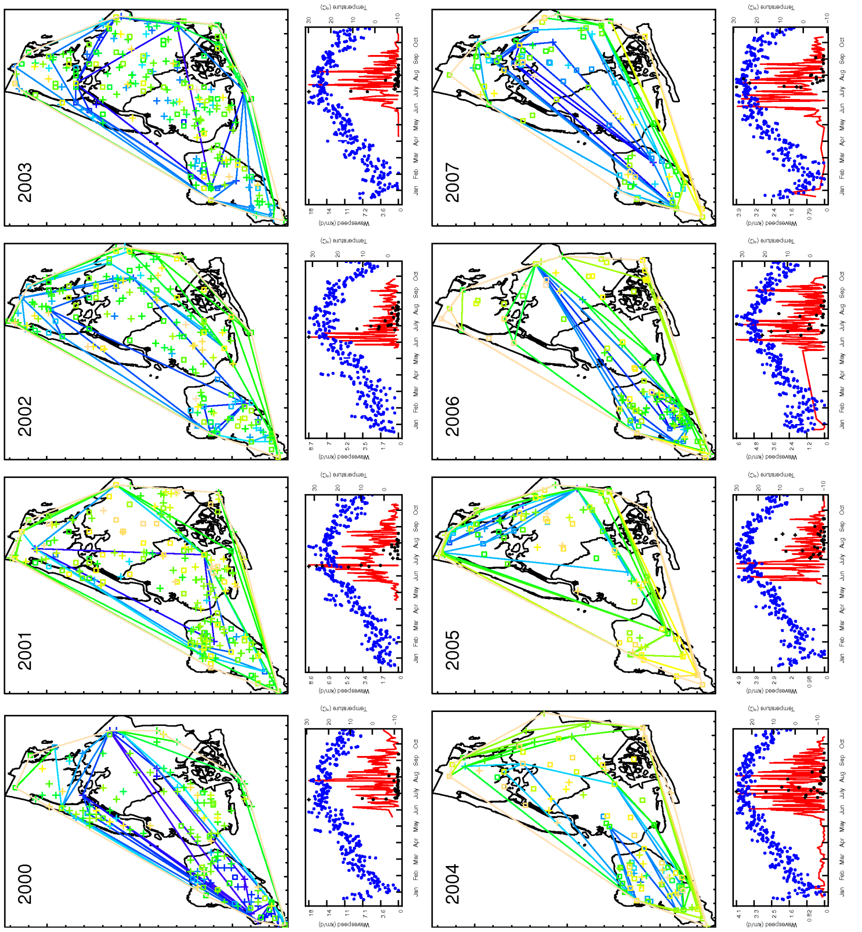

Figure S 1: Speed of WNV spread in NYC based on WNV-positive dead birds and WNV-positive mosquito pools reported to the NYCDOHMH. For each year, top subplots show the locations of WNV-positive dead birds (crosses) and WNV-positive mosquito pools (circles) collected in NYC. Colour coded convex hulls, ranging from blue to yellow, represent the estimated infected area at increasing dates. Bottom subplots show the estimated speed of the spread (change in infected area) of WNV in NYC (black), the mean daily temperature (blue), as well as the number of mosquitoes collected per day (red). See Fig. 1 of the main manuscript for further details.

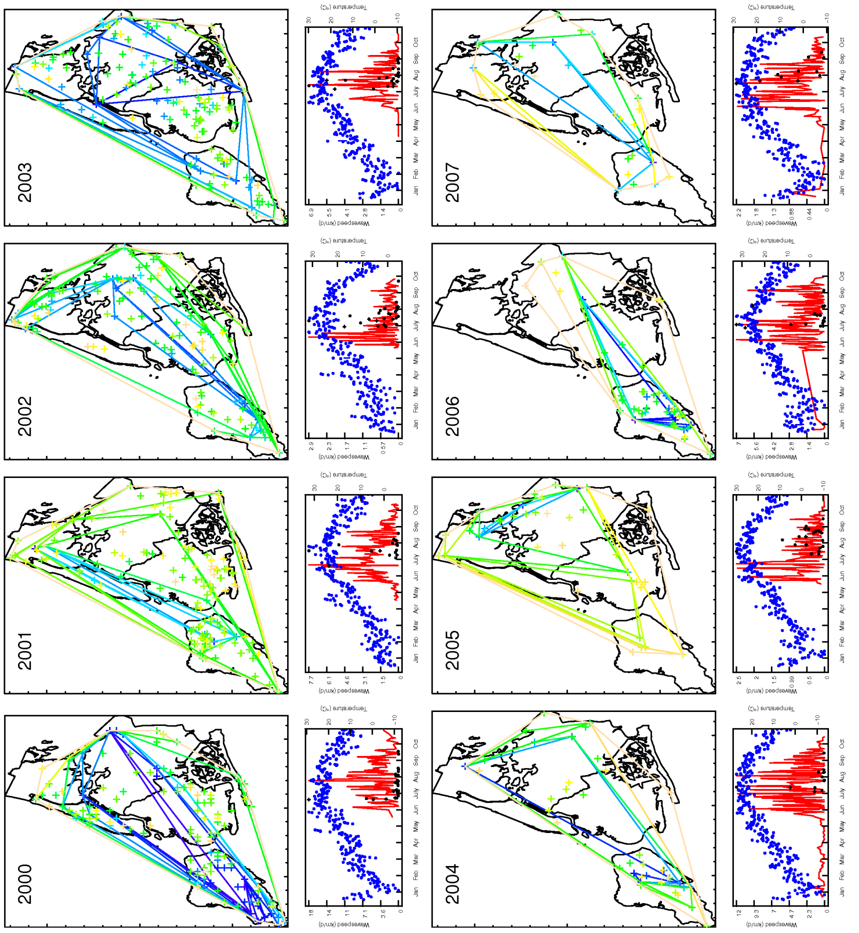

Figure S 2: Speed of WNV spread in NYC based on WNV-positive dead birds reported to the NYCDOHMH. For each year, top subplots show the locations of WNV-positive dead birds collected in NYC (crosses). Colour coded convex hulls, ranging from blue to yellow, represent the estimated infected area at increasing dates. Bottom subplots show the estimated speed of the spread (change in infected area) of WNV in NYC (black), the mean daily temperature (blue), as well as the number of mosquitoes collected per day (red).

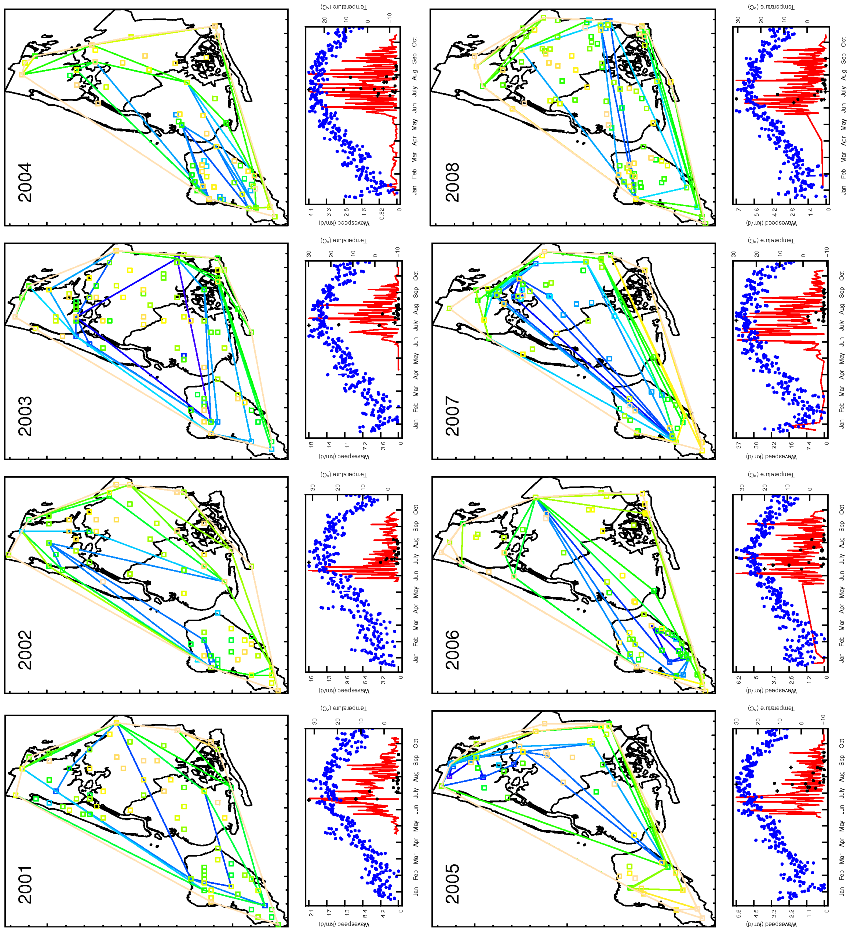

Figure S 3: Speed of WNV spread in NYC based on WNV-positive mosquito pools collected by the NYCDOHMH. For each year, top subplots show the locations of WNV-positive mosquito pools collected in NYC (squares). Colour coded convex hulls, ranging from blue to yellow, represent the estimated infected area at increasing dates. Bottom subplots show the estimated speed of the spread (change in infected area) of WNV in NYC (black), the mean daily temperature (blue), as well as the number of mosquitoes collected per day (red).

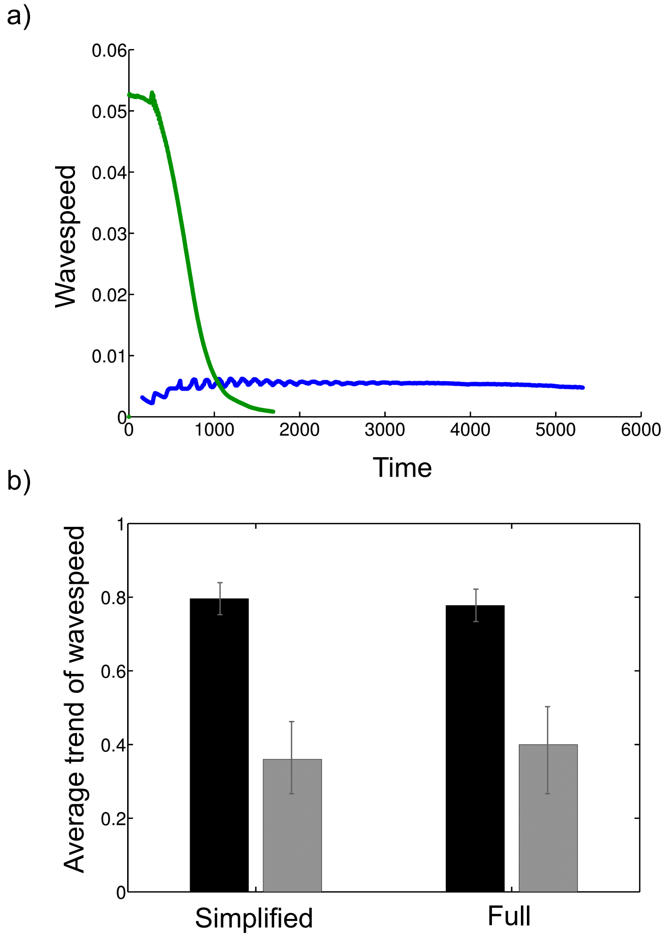

Figure S 4: Comparison of the simplified and the full WNV model. 100 realizations were run on a 100 x 100 heterogeneous (p=0.6) lattice for 6000 time-steps, with a dispersal rate of 0.01, a vector-to-host ratio of 1 and a detection threshold of 1%. (a) Average wave-speed measured with parameters in Table 2 for the simplified (blue) and the full model (green) shows that deceleration is accelerated by the high R_0 of the full model. (b) Average trend of wave-speed for the simplified and the full model. Black bars show the average ratio of the final wave-speed and the median wave-speed, error bars represent the standard error. Gray bars show the frequency of realizations with ratio (final/median wave-speed) above one, error bars show the 95% confidence interval. For a single site in the absence of dispersal, R_0 was calculated as 1.3925 for the simplified model and estimated as 1.7895 for the full model, by counting the number of secondary cases due to the introduction of a single infectious host.

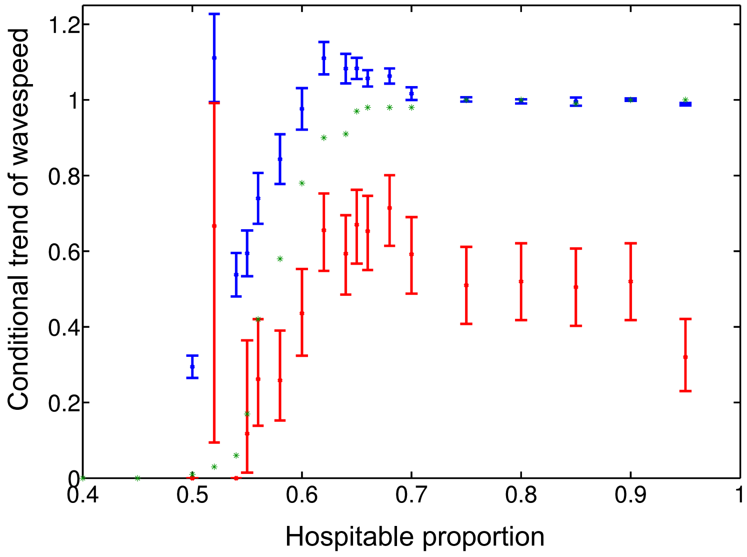

Figure S 5: Conditional wave-speed (blue), calculated as the average ratio of final and median wave-speed (ϕ), excluding failed realizations with zero final wave-speed. Parameters used are identical to Fig. 2d. Error bars show the standard error of the conditional trend. The frequency of realizations with $\phi > 1$ (red), error bars showing the 95% confidence intervals. Green stars represent the fraction of the realizations with non-zero final wave-speed. At $p = 0.52$, only 3 realizations reached the edge of the lattice, of which 2 had a ratio of final to median wave-speed above 1.

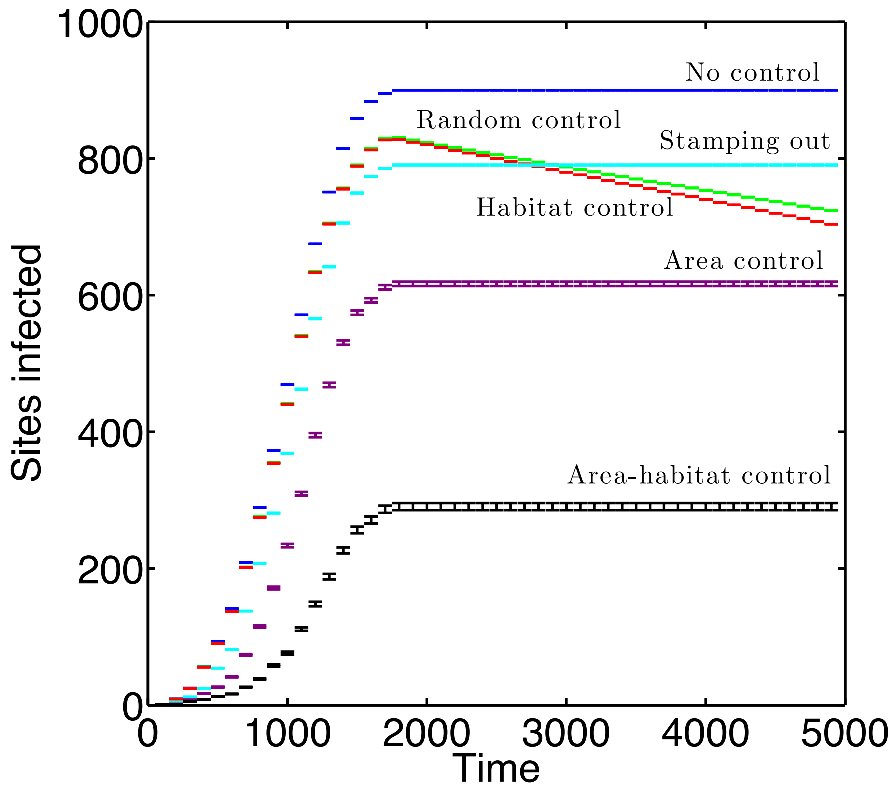

Figure S 6: A model-based control strategy targeting habitable sites in the vicinity of highly infected sites is more effective than uninformed control strategies even when all sites are habitable. Effectiveness is measured as the average number of sites where infectious hosts reached a threshold of 1%, with error-bars showing the standard errors (100 realizations; 30x30 homogeneous lattice (p=1.0); 5000 time-steps; dispersal rate of 0.01; vector-to-host ratio of 2). All control strategies made selected sites permanently inhospitable, but differed in their mode of selection. We depict the spread of WNV in the absence of control (blue symbols). Random control (green): random site every 25 time-steps (200 sites treated overall, 179.3 ± 0.3647 habitable sites treated); Habitat control (red): random transmission-promoting site every 25 time-steps (200 sites treated overall, all habitable); Stamping out (cyan): random site with $> 5\%$ infectious hosts every time-step (109.49 ± 0.4689 habitable sites treated overall); Area control (purple): random (Moore) neighbour of site with $> 5\%$ infectious hosts every time-step (548.53 ± 6.0744 sites treated overall, 283.46 ± 3.2305 habitable sites treated); Area-habitat control (black): random transmission-promoting (Moore) neighbour of site with $> 5\%$ infectious hosts every time-step (609.47 ± 5.3062 sites treated overall, all habitable).
